# Multi-epitope-based vaccine models prioritization against Astrovirus MLB1 using immunoinformatics and reverse vaccinology approaches

**DOI:** 10.1016/j.jgeb.2024.100451

**Published:** 2024-12-16

**Authors:** Awais Ali, Syed Luqman Ali, Abdulaziz Alamri, Elham Mohammed Khatrawi, Aliya Baiduissenova, Fatima Suleimenova, Vipin Kumar Mishra, Asifullah Khan, Marat Dusmagambetov, Gulsum Askarova

**Affiliations:** a Department of Biochemistry, Abdul Wali Khan University Mardan (AWKUM), Mardan 23200, Pakistan; b Department of Biochemistry, College of Science, King Saud University, Riyadh 11451, Saudi Arabia; c Department of Medical Microbiology and Immunology, Taibah University, College of Medicine, Madinah 42353, Saudi Arabia; d Department of Microbiology and Virology, Astana Medical University, Astana city 010000, Kazakhstan; e Department of Human Anatomy, Astana Medical University, Astana 010000, Kazakhstan; f Chemistry Division, School of Advance Sciences and Languages, VIT Bhopal University Bhopal, India; g Department of Dermatovenereology, Kazakhstan Medical University, Almaty, Kazakhstan, 050016

**Keywords:** Human Astrovirus MLB1, Reverse vaccinology, Immunoinformatics, Acute gastroenteritis, Vaccine designing

## Abstract

Astrovirus MLB1 (HAstV-MLB1) is non-enveloped RNA virus that cause acute gastroenteritis infection. Despite research progress about infection and pathogenesis of HAstV-MLB1, Currently, no vaccine has been developed to effectively combat this pathogen. The current study is based on immunoinformatics and reverse vaccinology approaches to design next-generation, multi-epitope-based vaccine models against HAstV-MLB1. Genome-wide whole proteome data of HAstV-MLB1 strain was retrieved, and a series of analyses were conducted to explore effective B and T-cell epitopes that hold significant antigenic nature with no toxicity and allergenicity. A set of vaccine constructs were designed by different combination of lead B and T-cell epitopes with diverse linkers and adjuvants sequences. The model vaccine structures were analyzed via rigorous criteria of physiochemical properties, antigenicity, and molecular docking with HLA and TLR4 immune receptors to ensure their efficacy and safety. Based on the lowest binding energy of −82.48 kcal/mol against the HLA receptor, the MLB1-C2 vaccine model with β-definsin adjuvant was prioritized for molecular dynamic and immune simulations analyses to assess its stability and immunogenic potential. These analyses revealed that the MLB1-C2 construct has feasible molecular stability and potential to boost strong immune responses in the host cell. Besides, the model was predicted to be non-toxic, non-allergenic, and antigenic, ensuring broad population coverage and capable to elicit a robust immune response. The *in-silico* cloning analysis highlighted a possible gene expression potential of the MLB1-C2 construct in *E.coli* commercial recombinant vector molecule. The findings of the current study provide an essential template for the development of a advanced next-generation effective vaccine against HAstV-MLB1.

## Graphical abstract

**Figure:**
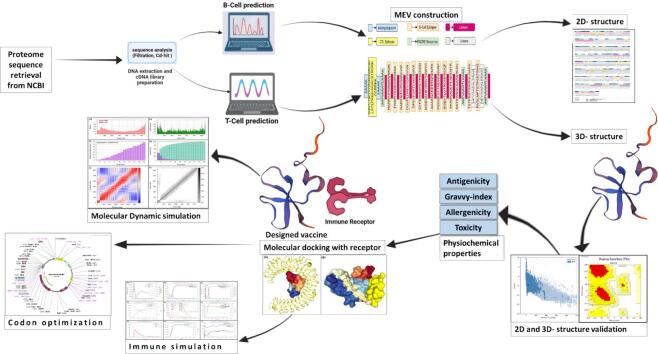

## 1 Introduction

Astroviruses were first isolated and characterized from human samples in 1975. Astroviruses are non-enveloped entities with a single-stranded, positive-sense RNA, wield their impact across an array of hosts spanning humans, turkeys, cattle, and beyond.^1^ Human astroviruses (HAstVs) cause acute gastroenteritis (AGE) in children globally. Besides, HAstVs responsible for other infections including, watery diarrhea that may last from two to four days, and less frequently headache, vomiting, fever, anorexia, and abdominal pains in children under the age of 2 years.^2^ HAstVs are reported to exhibit high-level genetic diversity and several recombinant strains with diverse and frequent pattern of recombination result in emergence of new HAstVs variants.^3^ A distinctive variant, i.e. Astrovirus MLB1 (HAstV-MLB1) emerged in 1999 from Japan^4^. HAstV-MLB1 is linked to acute gastroenteritis with over 1290 reported cases in infants, having age less than 6 months.^5^ Higher divergence of HAstV-MLB1 compare to other classical HAstVs has been reported.^6^ A seroprevalence study demonstrated a particular high infection occurrence of HAstV-MLB1 strain among children, suggesting the HAstV-MLB1 as primary cause of infection during childhood^4^. In addition, the HAstV-MLB1 prevalence has been reported beyond the gastrointestinal and possibly infect other tissues as well^7^. Despite a wide-spread infection and serious public health burden, still there is no vaccine-based therapies are available against HAstV-MLB1.^2^

In recent years, the landscape of vaccine designing has undergone a transformative shift, owing to the groundbreaking advancements in bioinformatics and cutting-edge Omics-based clinical discoveries^8^, ^9^. This paradigm shift has led to remarkable success in generation of next-generation, multi-epitope vaccines, as evidenced by the numerous discoveries achieved so far.^10^, ^11^, ^12^, ^13^ In light of these exciting achievement, the current study embraced the potential of bioinformatics and immunoinformatics approaches to predict suitable antigenic epitopes capable to mediate immune responses against HAstV-MLB1. By leveraging the predictive power of these methods, we designed a highly effective vaccine candidate molecule. The comprehensive analyses pursued in current study encompassed structural, immunological, and chemical characteristics that ensure the designing of robust and effective vaccine constructs against HAstV-MLB1 strain.^14^, ^15^ The objective of current study is to predict a novel multi-epitope vaccine (MEV) designs with aim to address the challenges posed by HAstV-MLB1 strain and to offer a potential solution to combat this infectious agent.

## 2 Methodology

### 2.1 Retrieval and analysis of HAstV-MLB1 proteins

**Fig. 1:**
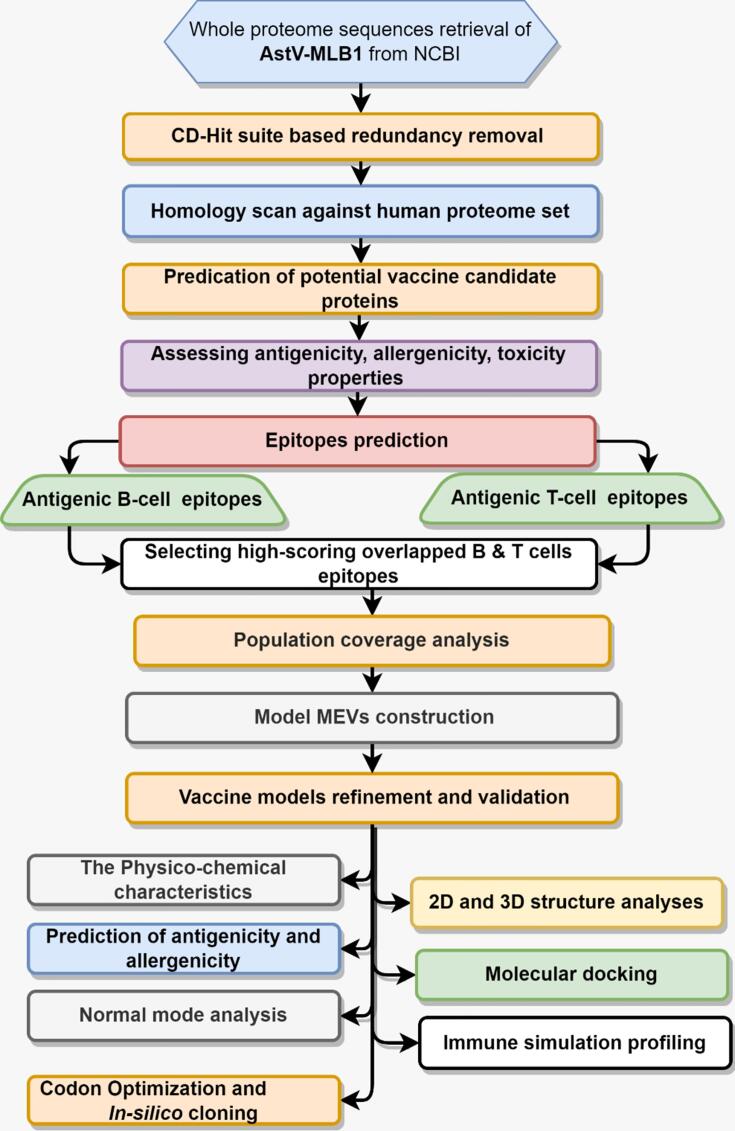
Methodological flow chart of the current study and strategies applied for the designing a multi-epitope vaccine designing against HAstV-MLB1 virus.

A schematic methodological layout of the present study has shown in Fig. 1. Entire proteome set of HAstV-MLB1 strain was obtained from the NCBI with taxonomy ID: 568715. Redundancy was removed through CD-HIT analysis by setting 85 % sequence identity threshold to obtain unique sequences. The resemblance between host and pathogen epitopes can pose a risk of autoimmune diseases due to potential cross-reactivity and molecular mimicry.^16^, ^17^ The non-redundant proteins were comparatively scanned against human proteome dataset via BLASTp, with specific threshold of; E-value ≥ 10^-4^, <50 % sequence identity, and <70 % query coverage.^18^, ^19^ The HAstV-MLB1 proteins which were non-homologues to human proteome were further examined for allergenicity, antigenicity, and toxicity features.

### 2.2 Immune cells epitopes selection

MHC-I and MHC-II epitopes were predicted using the corresponding tools in the Immune epitope database (IEDB).^20^, ^21^ FASTA sequences of pathogen vaccine candidate’s proteins were submitted to resource with human host specification. The resource employs SMM-align method to reproduce optimal IC_50_ value for each peptide sequence. The IEDB server assesses peptide affinity for MHC molecules through IC_50_ scores. IC_50_ < _50_ nM indicates strong binding, while values between 50–500 nM represent moderate to weak binding interactions. In this study, an IC_50_ threshold of < 100 nM was used to select epitopes with either strong (IC_50_ < 50 nM) or moderate binding affinity (50–100 nM), thus capturing the most relevant candidates for further analysis.^22^ For B-cell epitopes prediction, the ABCPred-2.0 webserver was utilized (accessed on July 15, 2023). The ABCPred prediction method uses both RNNs and conventional FNNs to make accurate predictions about B-cell epitopes. Next, the MHC-I, MHC-II common epitopes that overlapped with B-cell epitopes were prioritized in downstream analysis (Table S2).^23^

### 2.3 Assessment of immunogenic potential of selected epitopes

The epitopes have to fill full certain criteria to be a constituent of an effective next-generation vaccine construct. It should be antigenic to trigger an immune response, non-homologous to the host proteome, non-allergenic and non-toxic. An array for immunoinformatics resources, including VaxiJen, AllerTop, and Toxinpred were employed in current study to assess these characteristics of selected epitopes.^24^, ^25^

### 2.4 Population coverage and epitopes conservancy analysis

**Fig. 2:**
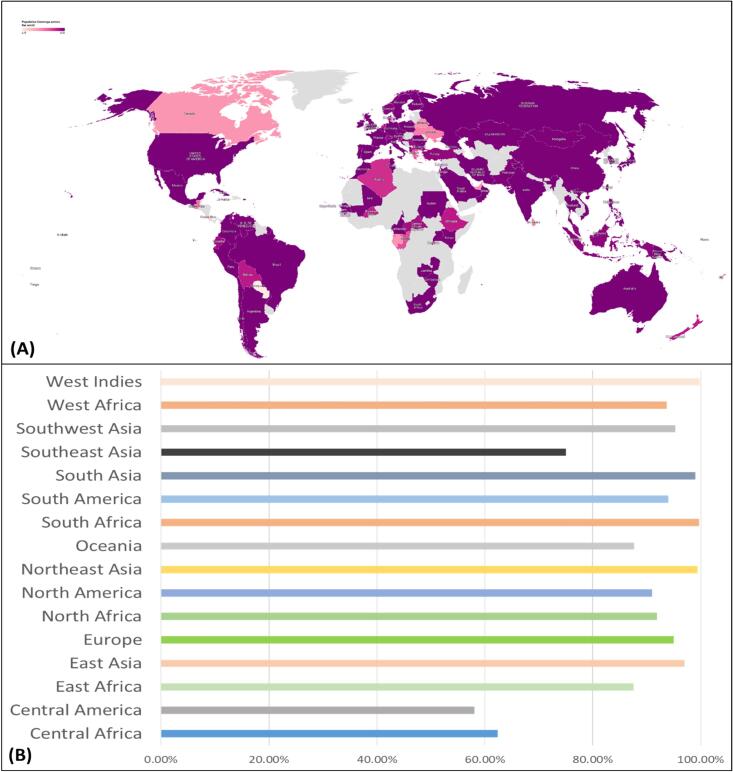
Population coverage evaluation of selected epitopes, (A) MHC-1 and MHC-2 epitope coverage for proteins across global regions. West- indies, East Asia, Europe, and South America show the highest overall coverage, (B) MHC-1 and MHC-2 epitope coverage for proteins across major continental ethnic groups.

Human populations belong from diverse ethnicities show variations in HLA alleles expression. This varied distribution of HLA strongly impact the vaccine efficacy among different ethnicities. The epitopes conservancy is therefore important to examine the degree of similarities among prioritized epitopes. We utilized the epitopes population coverage utility implemented in IEBD resource to examine the prioritized CTL, HTL epitopes binding potential with HLA alleles of diverse ethnicities (Fig. 2; Table S3).^26^ The IEBD resource calculates the T- cell epitopes population coverage based on HLA genotypic frequencies^27^, MHC binding, and T cell restriction data. This innovative approach gauges the fraction of individuals likely to respond to a specific epitope or epitope set, providing a comprehensive understanding of population coverage dynamics.^20^, ^28^, ^29^, ^30^, ^31^

### 2.5 Multi-epitopes vaccines (MEVs) construction

The top-ranked selected epitopes from HAstV-MLB1 vaccine candidate proteins were utilized in designing MEV models. Specified linkers, i.e. EAAAK, GGGS, HEYGAEALERAG and PADRE sequence were strategically incorporated between epitopes in model construct to enhance vaccine molecules expression levels and perform proper folding. The Top epitopes were connected to four distinct adjuvants: HBHA conserved peptide sequences HBHA, 50S ribosomal protein L7/L12 adjuvants, β-defensin, and protein, at the N-terminal, alongside the EAAAK linker to improve immunogenicity. Several MEVs models were designed from different combination of epitopes, linkers and adjuvants sequences and examined for safe and effective immunogenic potential. Four MEVs constructs were prioritized based on their allergenicity, toxicity and antigenicity features.^24^, ^25^

### 2.6 Assessment of immunological and physiochemical characteristics of MEVs

The physiochemical and immunological characteristics of the model vaccine designs were assessed using various web-servers. The physicochemical properties were assessed using the ProtParam webserver (ExPASy, 2020) from ExPASy^32^. Safety profiles of the constructs were examined using AllerTOP2.0 webserver by probable allergenicity estimation.^24^ Antigenicity was ascertained using the VaxiJen v2.0 webserver with a cutoff ≥ 0.4.^25^ Toxicity evaluation was performed using the ToxinPred 3.0 webserver.^33^ Further, the SOLpro server (https://scratch.proteomics.ics.uci.edu/) was applied to predict the possibility of protein dissolution when overexpressed in E. coli,^34^ SignalP 4.1^35^ and TMHMM v2.0102 servers were used to check any transmembrane helices in the vaccine and the existence of any signal peptides.^36^ By employing these servers, a comprehensive assessment of the vaccine's potency and safety was performed.

### 2.7 2D and 3D structures prediction, refinement, and validation

PSIPRED 4.0 webservers was used to forecast the secondary structure of the finalized MEV.^37^ The three-dimensional structure inferences of vaccine construct is vital to examine a vaccine's molecular interactions with immune receptors.^38^ AlphaFold Protein Structure database, an AI-powered website, predicted the 3D structure^39^, with later confirmation from SAVES v6.0 via the ERRAT server.^40^, ^41^ Procheck server was utilized to calculate Z-score and perform Ramachandran plot evaluation of the vaccine models to validate their structural refining.^42^, ^43^

### 2.8 Molecular docking with human immune receptors

The vaccine molecule causes a strong immunological reaction when it interacts with host immune cell receptors. The HAstV-MLB1 model vaccine's binding affinity with human immunological receptors was evaluated via molecular docking inspection. The Toll-like receptors (TLRs) belong to a family of pattern recognition receptors which are important for human defense against external infections agent. Among these the TLR3, TLR4 and TLR8 receptors are particularly important in mediating immune responses.^44^ Besides, the human leukocyte antigens receptors (HLAs) produce humoral response against antigen. The designed MEVs were docked against TLR3, TLR4, TLR8 and HLA (Pdb ids: 2a0z, 4G8A, 3w3m, and 5WJL) molecules using the Hawkdock server that performs protein–protein docking based on geometric shape complementarity.^45^ The docking results were subsequently validated from Hdock server.^46^ in the last for top priotized complex we have utilized the HADDOCK 2.4 platform to model and refine the complexes, analyzing key parameters such as van der Waals energy, electrostatic energy, desolvation energy, and RMSD.^47^

### 2.9 IMODS based molecular dynamic simulation assessment

Molecular dynamic simulation approach was followed to assess the stability of protein–protein complex. The IMODS webserver was used in current study to simulate the MEV-immune receptors complexes molecular stability in the cellular environment at 300 K constant temperature, 1 atm constant pressure at molecular mechanics level.^48^ Protein stability is calculated by comparing essential dynamics to their typical modes. The iMODS server provides a platform for normal mode analysis (NMA) that facilitate the assessment of typical protein mobility inside the internal coordinates.^48^ Furthermore, the webserver determined intrinsic movements using eigenvalues, covariance, B-factors, and deformability. The deformability of the main chain of vaccine candidate protein is defined, when a molecule deforms at each of its residues. The normal mode value reflects motion stiffness, with smaller eigenvalues suggesting simpler bending structures, which are directly proportional to the energy required for such deformations.

### 2.10 Molecular dynamics (MD) simulations

To assess the interactions between the vaccine and the receptor, we performed docking studies using AutoDock followed by 100 ns MD simulations.^49^ The MD simulations were conducted using AMBER18^1^ software with the ff14SB force field. The TIP3P water model was employed for solvation, and the system was enclosed in a 15 Å × 15 Å × 15 Å orthorhombic box^50^. The simulation protocol included a production phase of 100 ns, preceded by energy minimization, heating, and a 5 ns equilibration phase. The procedures followed the methodologies outlined in our previous publications (2–4). Trajectory analysis was performed using the CPPTRAJ^51^ module from AmberTools18, and visualization was accomplished with VMD.^52^ Image generation was done using PyMOL,^53^, ^54^ and data analysis was carried out with XMGrace, adhering to the established methodologies from prior studies.

### 2.11 Immune simulation analysis

The C-IMMSIM server performs immune simulation to examine the immune elicit capability of certain antigen molecule. The resource employs a machine learning-based method with position-specific scoring matrices (PSSM) for epitope prediction.^55^ C-IMMSIM server was employed to predicts the immunological capabilities of modelled MEVs by following the default parameters of the resource.

### 2.12 Codon optimization and molecular cloning prediction of MEV construct

Codon optimization was carried out using the Java Codon tool (https://www.jcat.de/) for the MEVs to obtain a more acceptable DNA sequence of the constructs.^56^ The molecular cloning potential of the optimized cDNA sequence of MEV in the *E. coli* (K12 strain) pET-28a (+) vector was examined using Snap-Gene software.^57^

## 3 Results

### 3.1 Astrovirus MLB1 proteins sequences retrieval, data compilation and homology scan against human host proteome

The 445 protein sequences, available in Genbank NCBI, for different samples of Astrovirus MLB1 strain were retrieved. The data was subjected to target prioritization and vaccine constructs designing (Fig. 1). The sequences set was compiled to remove redundancy and the non-redundant dataset of Astrovirus MLB1 proteins was homology scanned against human host entire proteome to obtain Astrovirus MLB1-specific and human host non– homologous targets. The analyses eventually identified five Astrovirus MLB1 vaccine candidate proteins, i.e. BAU68081.1, QQM16406.1, AKA09822.1, AKA09787.1, and AFJ68608.1. All these proteins were predicted by utilizing AllerTOP2.0, VaxiJen v2.0 and ToxinPred webservers, as highly antigenic, non-allergenic and capable to induce appropriate immunological responses (Table S1). These proteins were prioritized in downstream analyses.

### 3.2 Astrovirus MLB1 T and B cell epitopes prediction and population coverage analysis

The five key vaccine candidate proteins of Astrovirus MLB1 strain were subjected to a comprehensive analysis to pinpoint their essential epitopes. The T-cell epitopes (MHC-I and MHC-II) were predicted using the IEDB server with an IC_50_ threshold of < 100 nM, capturing both strong binders (IC_50_ < 50 nM) and moderate binders (IC_50_ between 50–100 nM). This approach focuses on peptides with strong and moderately strong binding affinities, prioritizing candidates for downstream analysis. Additionally, we utilized ABCpred to predict overlapping B-cell epitopes with significant scores > 0.7 and a specificity of 75 % (Table S2). The resultant epitopes were further examined to evaluate their potency in terms of low toxicity, heightened antigenicity, IFN-γ positivity, and a reduce risk of allergenic reactions all epitopes were non-allergen, Non-Toxin and Antigenic (Table 1). The selected epitopes demonstrated varying levels of global population coverage across different proteins. For MHC class I, the global coverage ranged from 94.90 % to 100 %, with proteins BAU68081.1 and AFJ68608.1 achieving 100 % coverage. For MHC class II, the coverage varied more widely, from 51.41 % to 100 % global coverage (Table S4) and significant coverage in regions that experiencing a higher prevalence of Astrovirus-MLB1, such as Northeast Asia, South Africa, West Indies, and the South Asia (Table S5; Fig. 2). The primary objective of these analyses was to identify lead epitopes, having broad population coverage and remarkable ability to trigger robust humoral and cell-mediated immune responses. These meticulous analyses identified three B and T cells overlapped lead epitopes for each of the prioritized proteins that were utilized in model vaccine constructs designing.

**Table 1:** Physicochemical and immunogenic properties of lead B-cell epitopes that internally overlapped with MHC-I and MHC-II epitopes sequences.

B-cell epitope	Antigenicity*	IFN-γ (positive Score)	Hydro-phobicity	Charge	Instability index (II)*
RKRRYIPNRNRRRRQN	0.6292	2.0	9	2239.8	24.38
TWEPIYADEGIPHRSA	0.5547	0.366	−1.5	1842.22	31.25
GARIHKDVRVGSNLVW	0.7603	0.207	2.5	1807.33	39.16
GGVPLDRPVYDFKVVN	0.5176	0.260	0	1775.27	96.88
DFEVFGPTVWDEIAYK	0.5231	−0.201	−3	1916.34	16.70
GFPDWSDPEYSSEEDD	0.9388	−0.700	−7	1875	31.2
MGEVAHKYERVYKWYC	0.4715	0.05	1.5	2062.62	29.37
AKARNADEPENDENTR	0.4996	0.137	−2	1830.06	25.62
EPARTIALHMANASTR	0.5103	−0.817	1.5	1739.2	27.31
PSGQFSTTMDNNMVNF	0.4405	3	−1	1790.18	7.31
SGEVTLQTRGNPSGQF	1.0200	1	0	1678.03	14.64
AKARNADEPENDENTR	0.4996	0.081	−2	1830.06	29.37
SSTSWSGLGARKHLDV	1.1490	1	1.5	1701.1	16.11
TSTPSSTSWSGLGARK	1.1384	1	2	1622.97	28.14
RVSLNPTSTPSSTSWS	1.1722	0.11	1	1707.04	31.65

* > 0.4 indicates antigenic potential.

*>40 classifies as the protein as stable.

### 3.3 MEV constructs designing

**Fig. 3:**
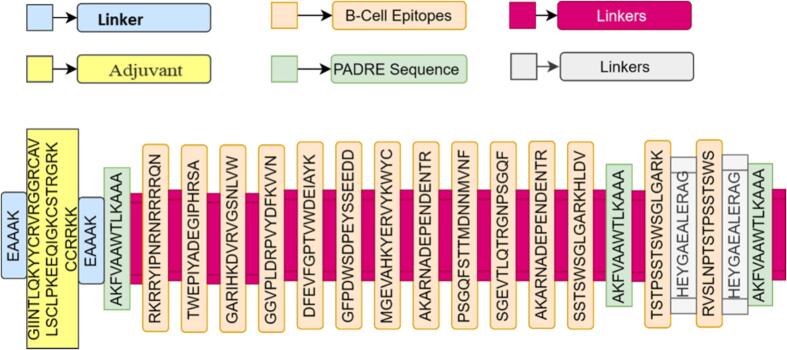
A MEV construct with β-definsin adjuvant by combining B and T cells epitopes with appropriate linkers.

The MEV constructs were designed from Astrovirus MLB1 selected epitopes, possibly recognized by CTL, HTL, and B cells, and possess high antigenicity, non-allergic properties, and non-toxic features (Table 1). These epitopes were joined together using different inkers like EAAK, GGGS, and HEYGAEALERAG. The linkers were used to limit the formation of junctional epitopes and improve epitope presentation during the vaccination (Table S5). For optimal adjuvant-MEV interaction, the lead epitopes, linkers and adjuvants were connected in such a way to ensure the stability, structural integrity and effective detachment of the protein components (Fig. 3; Table 3). This design possibly expands the MEV construct's potential and may provide optimal immunization.

### 3.4 MEVs construct assessment and validation

Four MEV constructs were predicted safe and effective based on their immunological assessment (Table 2). The antigenicity score > 0.5 ensured robust immunity, and significant antigenic potential. The designed vaccine constructs have molecular weights ranging from to 43,935 to 56,402 Da, and their hydrophilic nature was indicated by computed GRAVY rating between −0.108 to −0.325, aliphatic index values ranges from 48.22 to 60.89, indicating thermal stability and flexibility of model vaccines over a wide temperature ranges (Table 2).^58^ The solubility of the vaccine predicted upon overexpression was ranging fom 0.520 to 0.874 (Table S5). In addition, our designed vaccine constructs C1, C2, C3 and C4 does not encompass any signal peptides that would also specify or obstruct protein localization (Figure S10-S13). The TMHMM-2.0 server predicted that no production difficulties would be associated with expression(Figure S14-S17). The various adjuvants incorporation in constructs significantly affect the physicochemical features of the model vaccines. These results suggest that vaccine constructs may effectively trigger a robust immune response in human host and additional validation of their immune efficacy may worthy to investigate.

**Table 2:** Physiochemical and immunological properties of designed vaccine constructs.

Vaccine construct	No. of Amino Acid	Molecular weight (kDa)	Instability index (Significant > 30)	Theoretical PI	Antigenicity (Probable antigenic)	Grand average of hydropathicity (GRAVY)*	GC content (%)	CAI(0.85–1.0)	Aliphatic index
MLB1-C1 with HBHA adjuvant	536	56402.65	40.05	5.67	0.6024	−0.651	40.1	1.0	59.35
MLB1-C2 with β-definsin adjuvant	422	43935.2	36.05	9.38	0.6342	−0.714	40.0	1.0	48.22
MLB1-C3 with HBHA conserved adjuvant	527	55284.43	60.89	5.51	0.6006	−0.635	40.8	1.0	60.89
MLB1-C4 with Ribosomal protein adjuvant	507	52214.49	30.24	5.74	0.5819	−0.486	40.8	1.0	60.79

*Positive GRAVY value indicates hydrophobicity, while negative value indicates hydrophilic feature of the construct.

**Table 3:** Binding energy and docking score of MEVs constructs with immune receptors.

Vaccine constructs	TLR3 (2A0Z*)		TLR4 (4G8A*)		TLR8 (3W3M*)		HLA (5WJL*)	
	Binding Energy (kcal/mol)	Docking Score	Binding Energy (kcal/mol)	Docking Score	Binding Energy (kcal/mol)	Docking Score	Docking Score	Binding Energy (kcal/mol)
MLB1-C1	−13.0	−310	−27.96	−310.46	−52.89	−346.56	−301.66	−47.15
MLB1-C2	−42.14	−263.5	−82.48	−393.14	−43.8	−274.59	–323.26	−56.46
MLB1-C3	−12.23	−264.3	−35.5	−345.17	−21.85	−310.04	−374.47	−40.55
MLB1-C4	5.05	−207.3	14.23	−257.46	−19.95	−240.85	−254.46	1.43

* represents PDB ID of the receptor molecule.

### 3.5 Secondary and tertiary structure prediction, validation and refinement

**Fig. 4:**
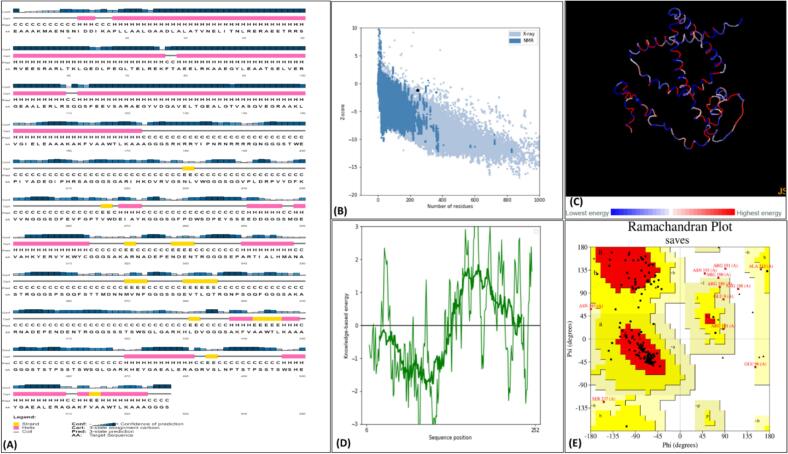
2D and 3D structure refinement and validation of MLB1-C2 (A) secondary structure of MLB1-C2 construct (B) the protein's overall quality, indicated by the Z-score of −1.2, (C) Tertiary structure energy map (D) ProSA-web plot showing residue scores in a native protein structure (E) Ramachandran analysis of model vaccine protein with 84.1% of residues in favored regions, 10.7% in the allowed region, 3.7% generously in allowed, and only 1.4 % in disallowed (1.4%) regions, indicating structural accuracy.

All the four constructs were proceeding for designing their protein structure models. Secondary structure analysis identified the presences of random coils, β-strands, and α-helices motifs in vaccine construct models indicating their well-balanced composition (Table S6). All the construct models exhibited > 80 % sequence identity to their respective PDB templates selected during 3D model generation (Figure S1-S4). Ramachandran plot analysis revealed significant number of models residues in allowed and favorable positions and the Z-score calculation for all models also depicted their high quality 3D structures generation (Fig. 4)(Figure S5-S7).

### 3.6 Molecular binding affinity assessment of vaccine constructs against TLRs and HLA types immune receptors

**Fig. 5:**
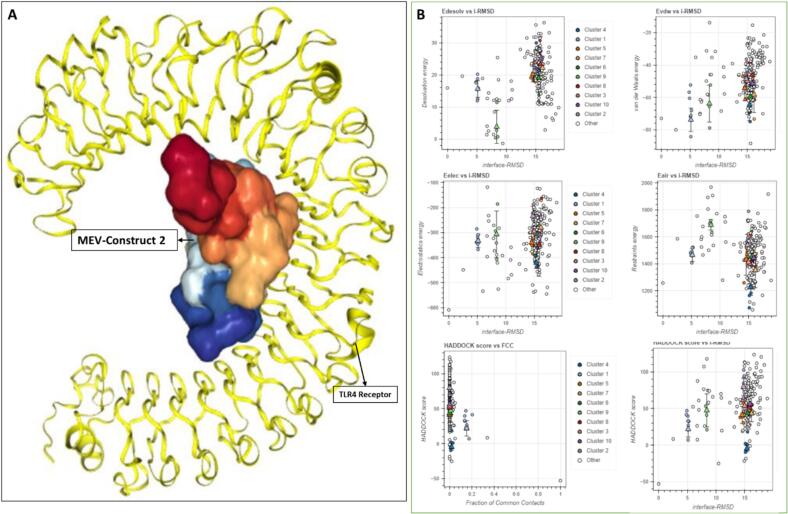
Significant molecular docking pose of lead anti-HAstV-MLB1 vaccine model (A) docked complex of MLB1-C2 with TLR4 (B) HADDOCK Cluster distribution for TLR4C2, showing the top 10 clusters. Cluster 4 exhibits the best HADDOCK score (−4.9 ± 3.8 kcal/mol) and a Z-score of −2.3, though with a higher RMSD (9.9 ± 0.1 Å) and significant restraints violation energy.

**Fig. 6:**
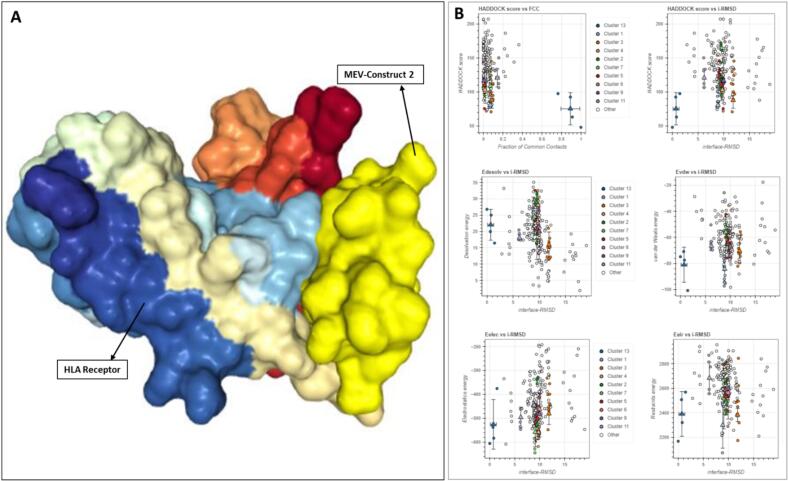
Molecular docking pose of lead anti-hastv-mlb1 vaccine model (a) docked complex of mlb1-c2 with hla (b) haddock cluster distribution for hlac2, showing the top 10 clusters. cluster 1 has the best haddock score (−6.2 ± 3.1 kcal/mol) and a Z-score of −2.5, indicating a reliable docking solution with low RMSD (3.5 ± 0.1 Å) and minimal restraints violation energy.

A molecular interaction between the immunological receptor's molecules and antigen's is important for an effective immune response generation. The molecular binding interaction of the vaccine models were examined via docking against human HLA, TLR3, TLR4 and TLR8 types of immune receptors. The binding affinity of the MLB1-C2 vaccine model was found top-ranked against human immune receptors. Especially in case of TLR4 and HLA receptors, the lead MLB1-C2 model developed robust molecular interactions as indicated by lowest binding energies, feasible docking scores and significant ACE values (Table 3; Fig. 5, Fig. 6).Further more these tope complexes were then assigned to HADDOCK for further assessment, In both cases, the Z-scores for the top clusters indicate reliable docking solutions, with MLB1-C2-HLA showing a more favorable overall binding energy profile (Z-score: −1.8) compared to MLB1-C2-TLR4 (Z-score: −2.3) (Fig. 5). Both complexes exhibit significant electrostatic interactions, with MLB1-C2-HLA having an electrostatic energy of −438.7 kcal/mol and MLB1-C2-TLR4 showing −420.8 kcal/mol (Fig. 5). These interactions contribute to their stability, although MLB1-C2-TLR4 demonstrates a higher RMSD of 9.9 Å, suggesting more conformational flexibility compared to MLB1-C2-HLA, which has an RMSD of 3.1 Å (Table 4) Figure.

**Table 4:** **HADDOCK Results for** MLB1-C2-TLR4 **and** MLB1-C2-HLA **Complex (Top Cluster)**.

Parameter	Value	
	MLB1-C2-TLR4	MLB1-C2-HLA
Cluster Number	4	2
HADDOCK Score	−4.9 +/- 3.8	−83.9 +/- 6.9
Cluster Size	6	6
RMSD from Lowest-Energy Structure	9.9 +/- 0.1 Å	3.1 +/- 0.2 Å
Van der Waals Energy	−64.7 +/- 8.4 kcal/mol	−67.9 +/- 4.9 kcal/mol
Electrostatic Energy	−420.8 +/- 18.8 kcal/mol	−438.7 +/- 19.2 kcal/mol
Desolvation Energy	21.4 +/- 4.2 kcal/mol	17.8 +/- 3.0 kcal/mol
Z-Score	−2.3	−1.8

### 3.7 Normal model analysis of lead MEV-C2 vaccine construct in complex with TLR4 (MEV-C2-TLR4)

**Fig. 7:**
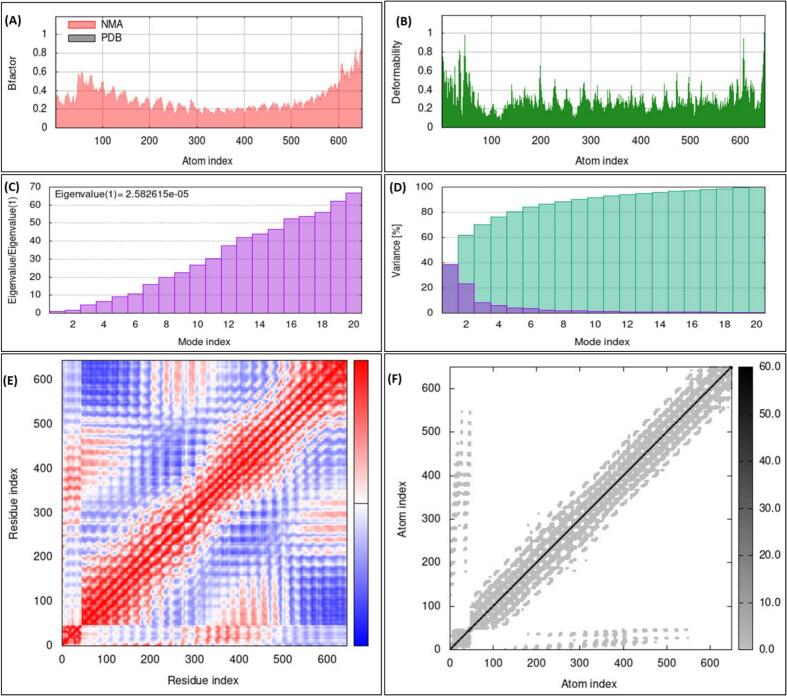
Significant structural flexibility of MLB1-C2-TLR4 complex showed during NMA analysis. The main chain deformability (A), quantified atom uncertainties with B-factor calculation (B) Deformability, the energy required to deform adjacent complexes. (C) Eigen values show a covariance matrix. (D) Residue correlations showing correlated (red), anti-correlated (blue), and uncorrelated (white) regions (E). The elastic network model demonstrated the interaction between springs and atoms, with darker grey denoting stiffer springs (F).

Normal mode analysis (NMA) of molecular dynamic simulation was followed via iMODS server to observe the stability and mobility of MEV-C2-TLR4 complex. The analysis predicted significant deformability of the complex, with each residue contributing in a different way to the complex flexibility. The Eigen value of 2.582615e-06 represent appropriate molecular stability of the complex. Each normal mode's variance was transformed into its eigenvalue to produce a B-factor value proportional to the RMS. The covariance matrix also revealed fascinating residue pair patterns, with related, unrelated, and anti-related motions in complex structure, being represented by blue, red, and white. Additionally, The elastic map revealed atom pairs intricately connected like springs, Showing variations in stiffness with darker grey areas denoting stiffer regions (Fig. 7A–E). This all results highlight the dynamic behavior and significant molecular stability of MEV-C2-TLR4 complex.

### 3.8 Molecular dynamic simulations analysis

**Fig. 8:**
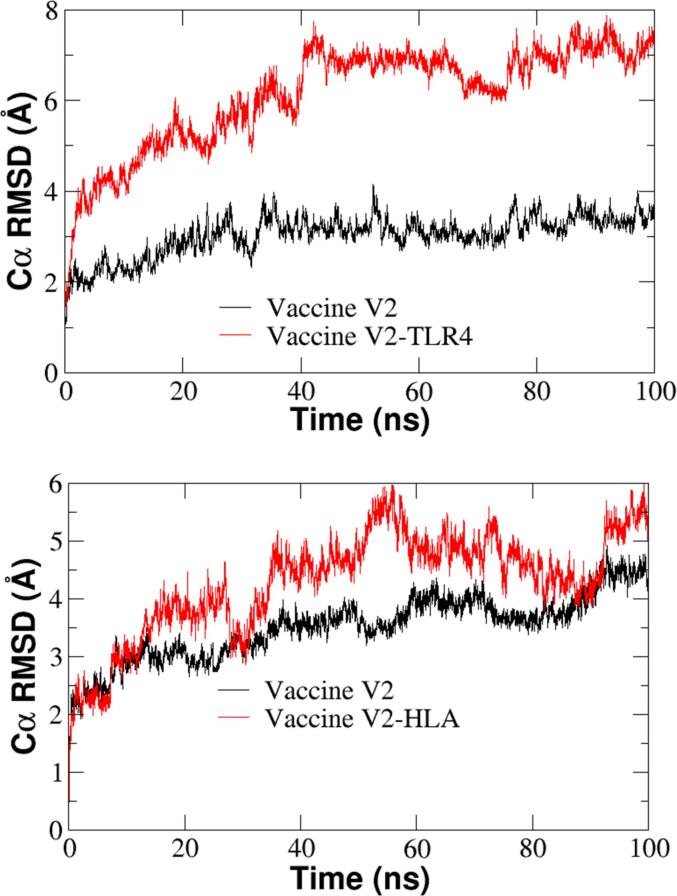
Time series of root mean square deviations (RMSD) for (A) HLA receptor with MLB1-C2, and (B) TLR4 receptor with MLB1-C2.

The molecular interactions between the vaccine and two distinct receptors were investigated using docking studies and molecular dynamics (MD) simulations. The compactness of the vaccine-receptor complexes was assessed through Root Mean Square Deviation (RMSD) and Root mean Square fluctuations (RMSF). The Cα Root Mean Square Deviations (RMSD) values indicated that the vaccine-TLR4 receptor complex (Fig. 8) exhibited higher flexibility compared to the vaccine-HLA complex (Fig. 8).

**Fig. 9:**
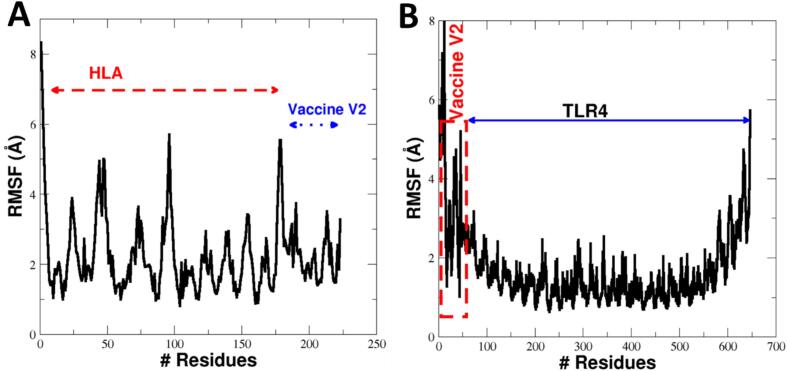
RMSF values for the (A) values for the MLB1-C2-HLA complex., and (B) MLB1-C2-TLR4 complex.

Despite this, the Cα-RMSD values for both complexes stabilized after 40 ns of simulation. Further analysis of the residue-specific fluctuations, as determined by RMSF values, revealed a maximum RMSF of 7 Å for the vaccine-Hal complex, while the vaccine-TLR4 complex reached a maximum of 8 Å (Figs. 9A and 9B).

**Fig. 10:**
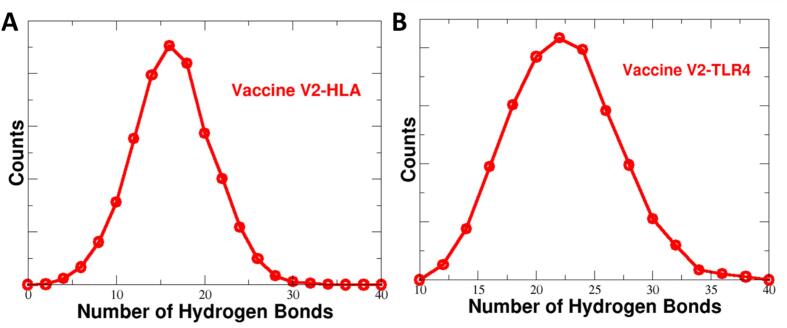
(a) The number of hydrogen bonds between the HLA receptor and the vaccine, (b) The Number of hydrogen bonds between TLR4 receptor and vaccine.

The terminal regions in both complexes displayed greater flexibility compared to the central regions. The 100 ns molecular frames were clustered into five distinct clusters. The most populated clusters are displayed in Figures S7 and S8. In evaluating the specific interactions between the vaccine and the receptors, key residue pairs were identified. For the vaccine-TLR4 complex, residues Arg43, Arg17 and Arg43 of the vaccine MLB1-C2 formed strong interactions with TLR4 receptor residues Leu423, Asp424 and Asp398 respectively (Table 5). However residues of HLA receptors Asp14, Asp32 and Asp163 make strong hydrogen bonds with residues Gln207, Arg192 and Thr213 of Vaccine MLB1-C2 respectively. These interactions are crucial for the stability of the vaccine-receptor complexes. The stability of the complex was further reinforced by the hydrogen bond analysis, which showed an average of 18 hydrogen bonds forming between the HLA receptor and the vaccine (Fig. 10A), compared to 22 hydrogen bonds observed between the TLR4 receptor and the vaccine (Fig. 10B). This high number of hydrogen bonds indicates a strong and stable interaction between the two components.

**Table 5:** Binding free energy components of TLR4 receptor and Vaccine MLB1-C2.

ΔE_ELEC_	−2627.08
ΔE_VDW_	−84.91
ΔEPB	2627.80
ΔEPB_np_	−12.75
ΔE_Disper_	0.0
ΔG(ΔH_PB_-TΔS)	−96 ± 2

To further quantify the overall stability of the MLB1-C2 comlpex, binding free energy calculations were performed using the Molecular Mechanics Poisson-Boltzmann Surface Area (MM-PBSA) method. Binding free energy (ΔG) of vaccine MLB1-C2- HLA receptor complex was calculated from the 100 ns simulation. The molecular-mechanical energy calculations were performed using MM/PBSA, and entropy calculations using nmode analysis. ΔE_EELEC_, ΔE_VDW_, ΔEPB_np_ and ΔEPB_solv_ are referred to the electrostatic, Vander Waals, polar, the non-polar contribution to the solvation energy and the electrostatic contribution to the solvation energy, respectively. This approach accounted for various energetic contributions, including van der Waals forces, electrostatic interactions, and solvation energy. The binding free energy for the vaccine-receptor complex (with TLR4) was calculated to be −96 kcal/mol, while with the HLA receptor, it was −92 kcal/mol, indicating strong and stable interactions in both cases (Table 5, Table 6).This negative free energy value reflects a highly favorable binding affinity and reinforces the overall stability of the complex.

**Table 6:** Binding free energy components of HLA receptor and Vaccine MLB1-C2.

ΔE_ELEC_	−2616.50
ΔE_VDW_	−100.59
ΔEPB	2636.28
ΔEPB_np_	−12.58
ΔE_Disper_	0.0
ΔG(ΔH_PB_-TΔS)	−93 ± 2.4

Overall, these findings emphasize the importance of the vaccine's molecular interactions with both TLR4 and HLA receptors. The stable and favorable interactions observed suggest the potential for enhanced vaccine efficacy and receptor engagement, which could play a crucial role in improving immune responses and vaccine effectiveness.

### 3.9 Immune simulation assessment of lead MLB1-C2 vaccine model

**Fig. 11:**
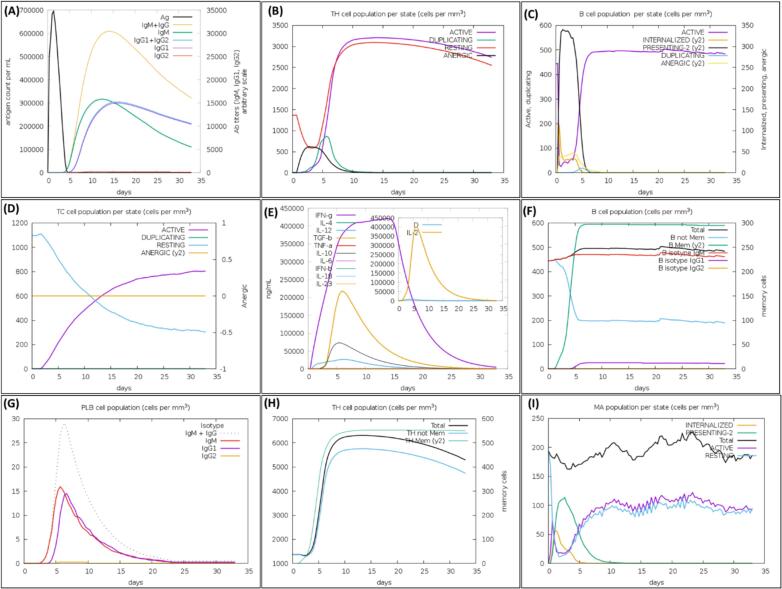
The C-ImmSim web-server was used to assess the immune responses to the lead MLB1-C2 vaccine construct, demonstrating dynamic immune changes over time. (A) After antigen stimulation, primary B-cell antibodies (IgM + IgG) increased, forming an essential barrier against infections. (B) Changes in the HTLs' secretion levels show a strong cellular immune response. (C) Increased secretion from active B cells further boosted the immune response. (D) Levels of CTL secretion increased, indicating successful targeting of infected cells. (E) An important finding was that levels of cytokine secretion, primarily IL-2 and IFN-**γ**, increased, demonstrating the vaccine's potential to activate critical immune mediators. (F) After the 3rd dose, B-cell count shows a remarkable increase, leading to a dynamic switch between various B-cell isotypes. This leads to a distinct accumulation of active plasma B cells producing both IgM and IgG antibodies). (G-H) a robust response is observed among the active phase T-cell population, showing higher concentrations compared to the anergic phase T-cellss (I) showing macrophage activation.

The C-ImmSim server was used to mimic the immunological response of the MLB1-C2 vaccine model, assessing its effectiveness and capabilities. The model vaccine construct is anticipated to provoke a robust innate immune response. Besides, It was found that the lead vaccine construct stimulate B cells to generate suitable amounts of IgG and IgM antibodies (Fig. 11A), to establish immune memory response. The population of T-helper and cytotoxic T-cells were predicted to enhance with proposed MLB1-C2 vaccine dosage (Fig. 11B, D), displaying strong reactions and successful memory growth. The number of active cytotoxic T lymphocytes slowly increased and peaked 60 days during stimulation, whereas the number of resting cytotoxic T lymphocytes increased in the opposite direction (Fig. 11D). Additionally, the MLB1-C2 vaccine was predicted to significantly increase the number of active B lymphocytes and IFN-ɣ, IL-2 levels with repeated exposure dosage of proposed construct with four weeks apart, demonstrating strong immunogenicity of MLB1-C2 model (Fig. 11C, 11E). Active phase T-cells also respond robustly with higher concentrations compare to the anergic phase (Fig. 11F-H), Macrophage activation as per immune response showed upper trend (Fig. 11I). These results underline the lead MLB1-C2 vaccine capacity to elicit a robust and diverse immune response (see Fig. 11).

### 3.10 *In-silico* cloning assessment of lead vaccine construct (MLB1-C2)

The lead construct codons were optimized via Java Codon Adaptation Tool (JCat) to examine its maximum protein expression. The resulting optimized codon exhibited a CAI of 1.0, complemented by an estimated GC content of 40.0 %. These findings suggest a stable vector expression in *E. coli*. Subsequently, the Snapgene tool predicted that the optimized codon sequence of lead MLB1-C2 vaccine construct predicted capable to feasibly clone in pET-28a (+) to generate a recombinant plasmid (Figure S9).

## 4 Discussion

Preventing HAstV-MLB1 outbreaks remains challenging. There is documented increase in human HAstV-MLB1 infection cases and occasional global clusters. Existing vaccines offer only moderate protection, particularly in young children and those with preexisting medical conditions^4^. Planning innovative treatment strategies against HAstV-MLB1 become indispensable now a day. Access to genomics and proteomics data and current advancements in reverse vaccinology and immune-informatics have greatly aided in vaccine development protocols.^59^, ^60^ Utilizing these cutting-edge resources are evidenced more effective compare to conventional methods of vaccine development. Epitope-based vaccines stand out as a promising approach, and offering superior safety and efficacy. Embracing these advancements are critical in the ongoing fight against HAstV-MLB1 outbreaks.

Chimeric vaccines have been designed using immunoinformatics and vaccinomics techniques to protect against a variety of pathogens, including Malaria,^14^ Zika virus,^61^ HIV^62^, SARS CoV-2,^12^, ^63^, ^64^ Nipah virus^65^, Tuberculosis^66^, West Nile virus^67^, and Dengue.^68^, ^69^ These state-of-the-art analysis sketch promising template for designing vaccine candidates with potent immunogenic and antigenic properties. To stay ahead of emerging problems, it is also critical to continuously monitor strain evolution and evaluate epitope conservation and population coverage. Therefore, this strategy is most advanced to develop potent vaccines and combat persistent viruses.

The present research, a promising multi-epitope-based vaccine models were designed against HAstV-MLB1. All available protein sequences data of the HAstV-MLB1 was analyzed to identify potential vaccine targets. Total five immunogenic and safe vaccine candidate proteins were identified, and potent epitopes were predicted from these proteins based on strong immune response, non-toxic, and unlikely to cause allergies. To enhance the vaccine's effectiveness, we employed rigorous criteria to predict MHC-I, MHC-II and B-cell epitopes^70^. B-cells activate the humoral immune system, neutralizing viruses and creating a protective memory^71^, ^74^. However, this response can be less effective and diminishes over time^71^. On the other hand, T-cells (CTL, HTL) induce a cell-mediated immune response, restricting pathogen spread through cell destruction and antiviral cytokine secretion, providing lifelong immunity by developing memory cells.^72^, ^73^ The current vaccine design incorporates multiple B-cell, CTL, and HTL epitopes, along with linkers and β-defensive adjuvants to enhance immunogenicity. To optimize expression, enhance bioactivity, and elicit robust immunogenic responses, we employed EAAAK linkers to connect adjuvant sequences at the N-terminus of vaccine constructs. Additionally, HEYGAEALERAG and GGGS linkers, recognized for their flexibility based on Solanki & Tiwari, (2018), were used to connect the prioritized epitopes. The EAAAK linker served as a stiff spacer, ensuring a strong bond between the adjuvant's N terminus and the epitope. This strategic use of linkers contributes to the overall effectiveness of the designed vaccine (Table S5). Top-ranked vaccine models predicted during this study exhibited high antigenicity, while being safe. The top-ranked designed vaccine demonstrated molecular stability, basicity, and hydrophobicity that additionally support its potential to elicit a strong immunogenic response. After a successful refining process, the 3D structures of the proposed HAstV-MLB1 vaccine models were significantly improved, displaying desired stability (as confirmed by Ramachandran plot investigation). Studies have indicated the involvement of TLRs in recognizing viral peptide structures and activating the immune response.^31^, ^44^ Consequently, the molecular docking of the AstV −MLB1 vaccine models analysis was performed with TLR3, TLR4, TLR8 and HLA receptors. These results revealed promising biochemical interactions between the proposed vaccines construct models and TLRs, particularly in case of MLB1-C2 construct affinity with TLR4. The MLB1-C2 construct was prioritized due to its exceptional physicochemical characteristics, non-toxicity, and potential to boost both cell-mediated as well as humoral immunological responses. NMA and MD simulations predicted the significant molecular stability for MLB1-C2-TLR4 complex, which is required to elicit immune response. Immune simulations analysis demonstrated the potential of the top-ranked lead vaccine construct capability to elicit strong primary, secondary, and tertiary immune responses, with a notable increase in IFN-γ cytokine production, known for its effectiveness in combating viral diseases.^75^, ^76^ This theoretical vaccine model serves as a valuable tool for experimentalists to evaluate this model vaccine’s immunogenicity against HAstV-MLB1 infection and ultimately expedite vaccine designing strategy against this pathogen. While acknowledging limitations, such as the inherent uncertainties in prediction methods and challenges associated with immunoinformatics, the proposed vaccine constructs need experimental validation for safety. Recent studies about experimentally validation of the efficacy of such multi-epitopes vaccine constructs are inspiring in this regard.^77^, ^78^, ^79^, ^80^, ^81^ Additionally, the proposed vaccine models are based on top-ranked epitopes of HAstV-MLB1 strain and may therefore effective to combat only the HAstV-MLB1 mediated infection.

## 5 Conclusion

The emerging HAstV-MLB1 poses a serious public health concern. Promising multi-epitope vaccine models were designed against HAstV-MLB1 using cutting-edge immunoinformatics strategies. By incorporating T-cell and B-cell epitopes from the HAstV-MLB1 proteins, we aim to trigger strong cell-mediated and humoral immune reactions. The lead vaccine construct, i.e. MLB1-C2 showed encouraging results in terms of stable binding with the TLR4 receptor molecule during immunological simulations. In vitro and in vivo investigation may worthy to ensure the vaccine model efficacy against HAstV-MLB1. The proposed templates of the current study provide a roadmap to design an effective, next-generation anti-HAstV-MLB1 vaccine.

## CRediT authorship contribution statement

**Awais Ali:** Writing – review & editing, Writing – original draft, Software, Project administration, Methodology, Investigation, Formal analysis, Data curation, Conceptualization. **Syed Luqman Ali:** Writing – review & editing, Validation, Software, Resources. **Abdulaziz Alamri:** Writing – review & editing, Supervision, Investigation, Funding acquisition. **Elham Mohammed Khatrawi:** Writing – original draft, Software, Resources. **Aliya Baiduissenova:** Writing – original draft, Visualization, Validation, Formal analysis, Data curation, Conceptualization. **Fatima Suleimenova:** Visualization, Methodology, Investigation. **Vipin Kumar Mishra:** Writing – review & editing, Software, Resources, Project administration, Methodology, Investigation, Formal analysis. **Asifullah Khan:** Writing – review & editing, Supervision, Project administration, Conceptualization. **Marat Dusmagambetov:** Writing – review & editing, Project administration, Methodology, Investigation, Formal analysis. **Gulsum Askarova:** Writing – review & editing, Data curation, Conceptualization.

## Funding

Authors are thankful for Researchers Supporting Project number (RSPD2024R552), King Saud University, Riyadh, Saudi Arabia.

## Declaration of Competing Interest

The authors declare that they have no known competing financial interests or personal relationships that could have appeared to influence the work reported in this paper.
