# Supplementary material for: Multi-epitope-based vaccine models prioritization against Astrovirus MLB1 using immunoinformatics and reverse vaccinology approaches
Source: J Genet Eng Biotechnol. 2024 Dec 16;23(1):100451. doi: 10.1016/j.jgeb.2024.100451 (PMC11719404; doi:10.1016/j.jgeb.2024.100451)
Supplement: Supplementary Data 1 [file mmc1.docx]

**Multi-epitope-based vaccine models prioritization against Astrovirus MLB1 using immunoinformatics and reverse vaccinology approaches**

Awais Ali ^1†^, Syed Luqman Ali ^1†^, Abdulaziz Alamri^2*^, Elham Mohammed Khatrawi^3^, Aliya Baiduissenova^4^, Fatima Suleimenova^5^, Vipin Kumar Mishra^6^, Asifullah Khan^1*^, Marat Dusmagambetov^4^, Gulsum Askarova^7^

**Supplementary Figures:**

^
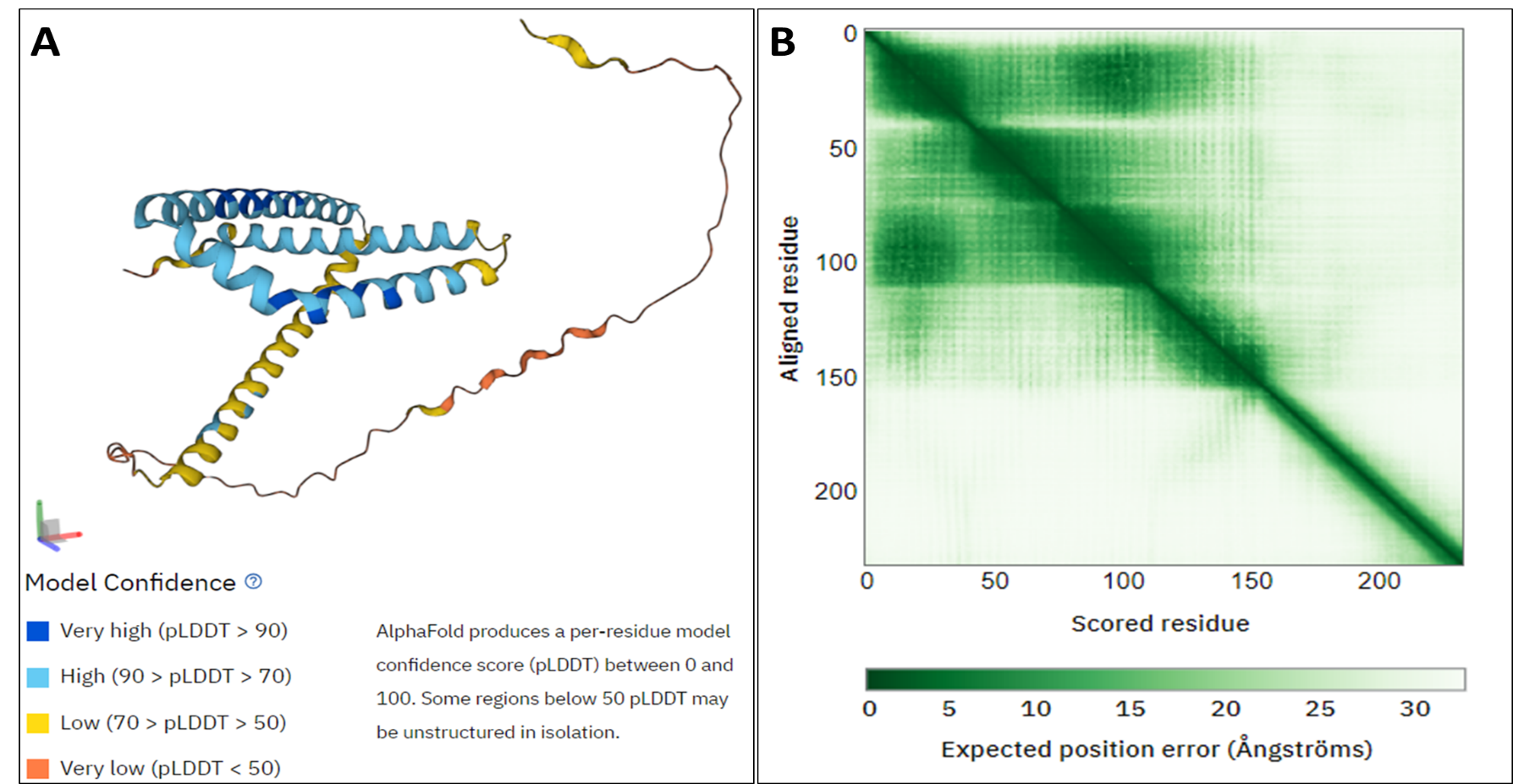
^

**Figure S1: (A)**3D structure of MLB1-C1 construct showing 80.0% sequence identify with AFDB:X7VFI6 (B) Predicted aligned error (PAE) assessing inter-domain accuracy.

**
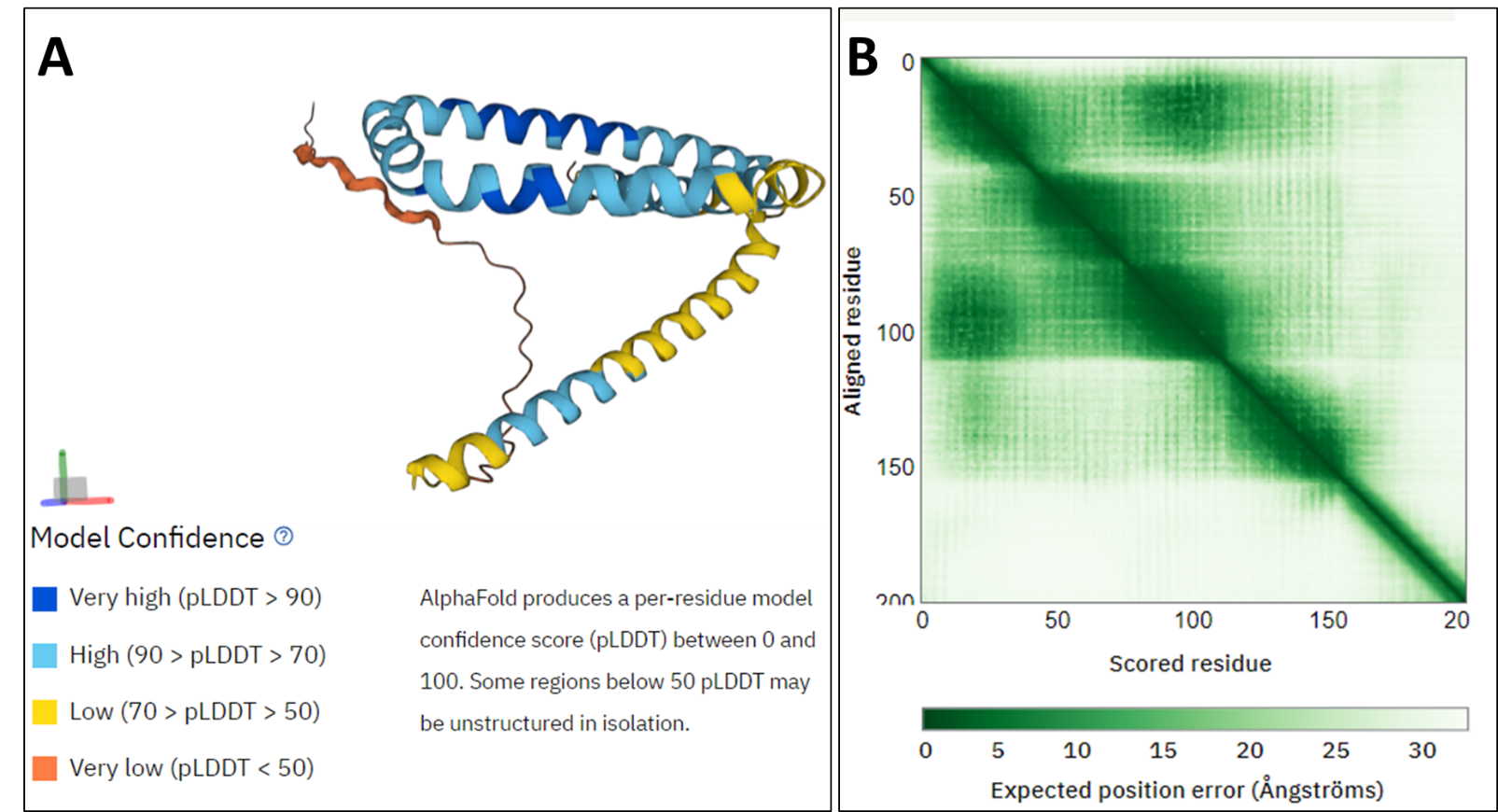
**

**Figure S2: (A)** 3D structure of MLB1-C2 construct showing 92.1% sequence identify with P81534.1. (B) PAE showing inter-domain accuracy.

**
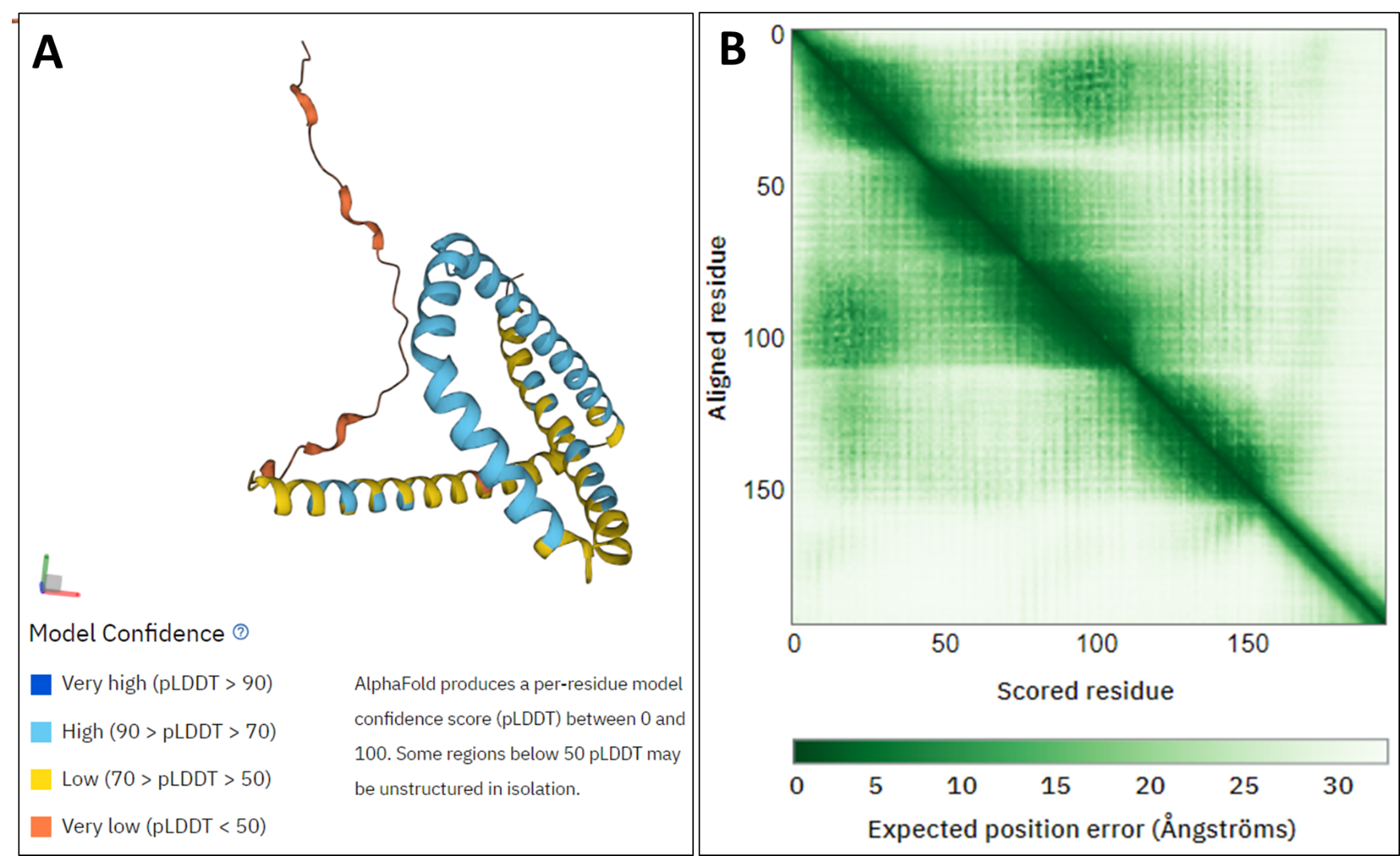
**

**Figure S3: (A)** 3D structure of MLB1-C3 construct showing 87.2% sequence identify with AFDB:A0A7M0C019 . (B) PAE presenting inter-domain accuracy.

**
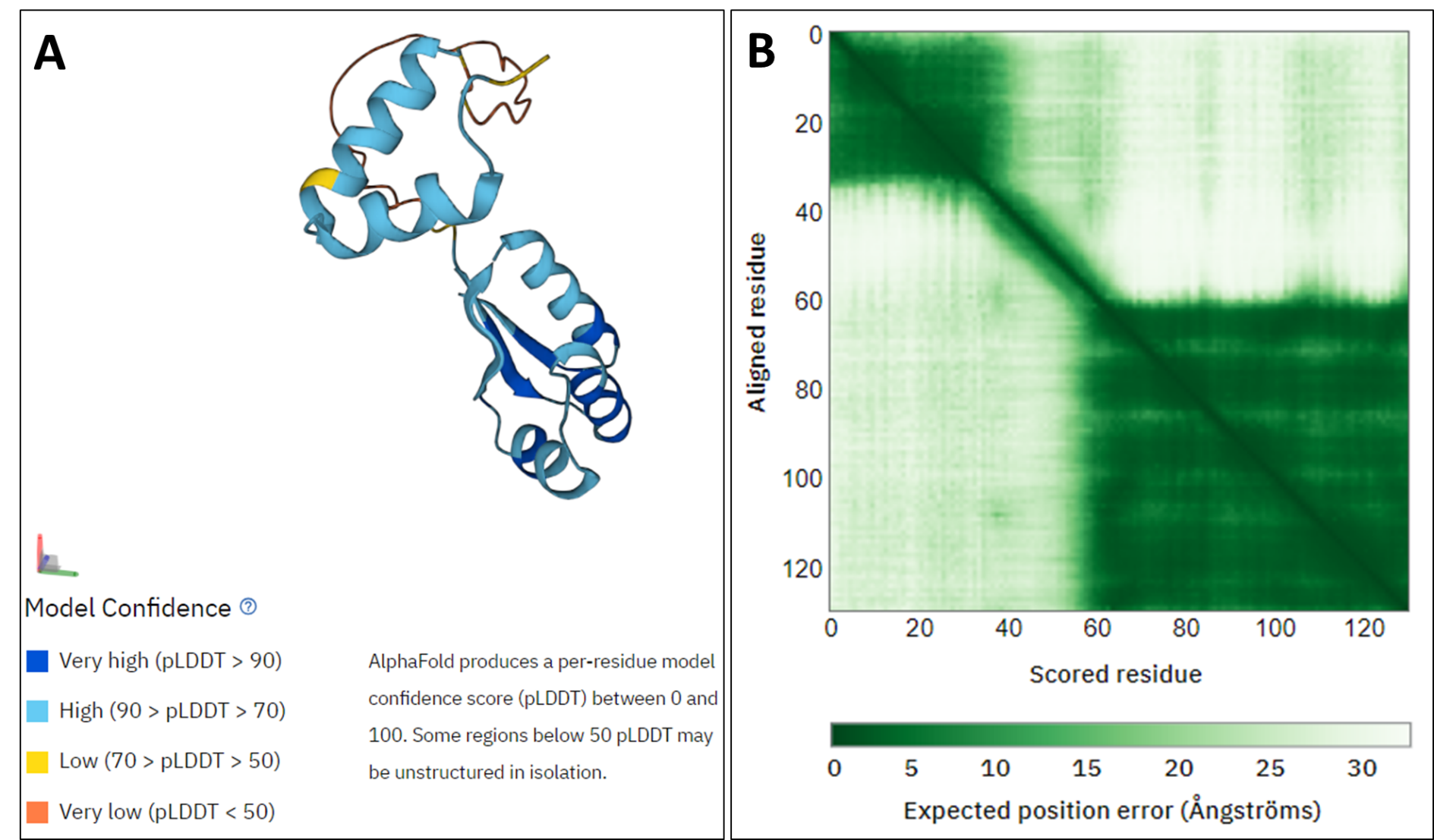
**

**Figure S4: (A)**3D structure of MLB1-C4 construct showing 100.0% sequence identify with AFDB:A0A0K2HSM3 (B) PAE shwoing inter-domain accuracy.

**
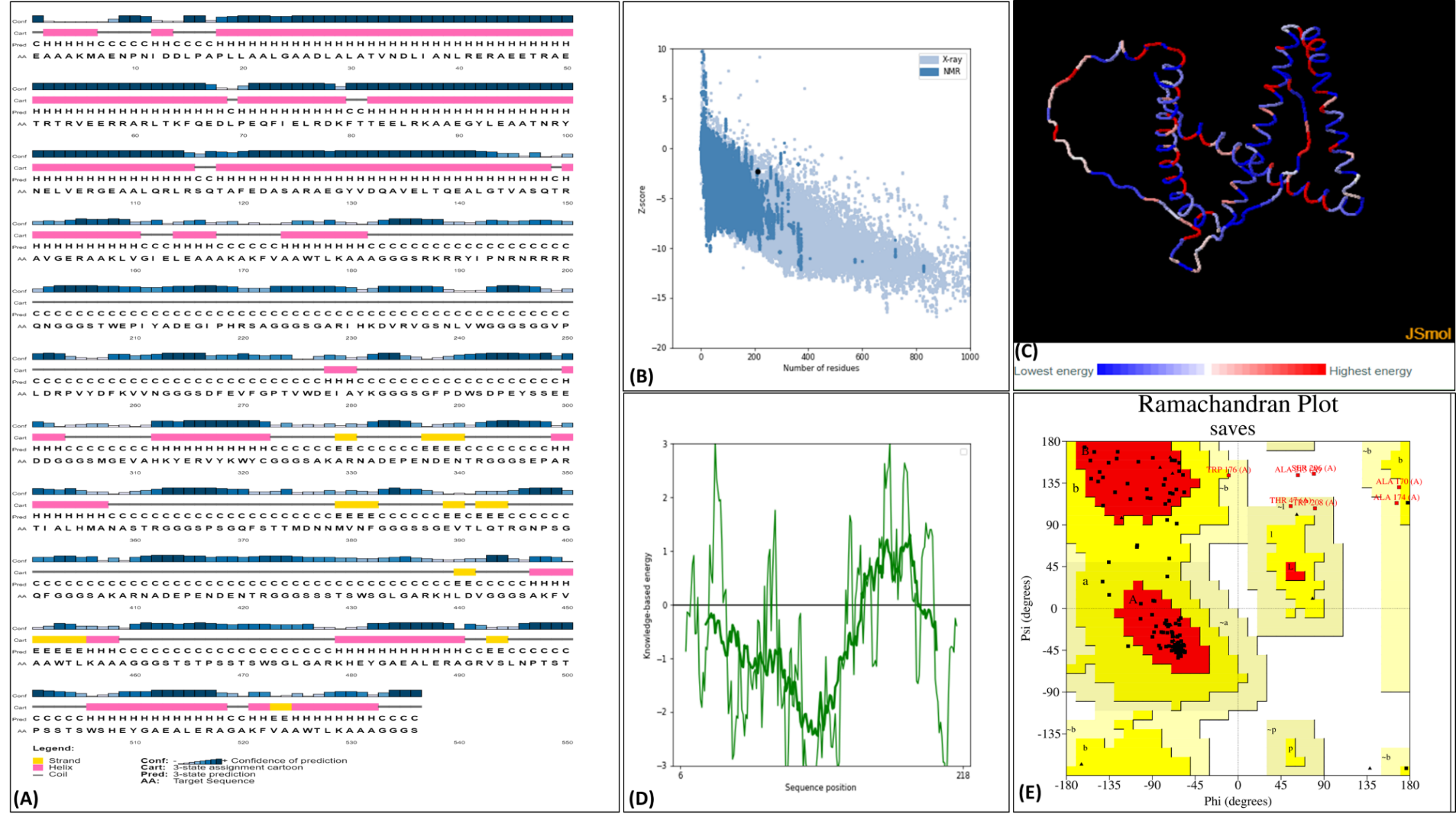
**

**Figure S5:** 2D and 3D structure refinement and validation of MLB1-C2 (A) secondary structure of MLB1-C2 construct (B) the protein's overall quality, indicated by the Z-score of -2.32, (C) Tertiary structure energy map (D) ProSA-web plot showing residue scores in a native protein structure (E) The refined vaccine protein displayed outstanding structural characteristics with 90.6% of residues in highly favored regions, 5.8% in the allowed region, generously allowed (2.6%), and disallowed (1.0%) regions in the Ramachandran plot.


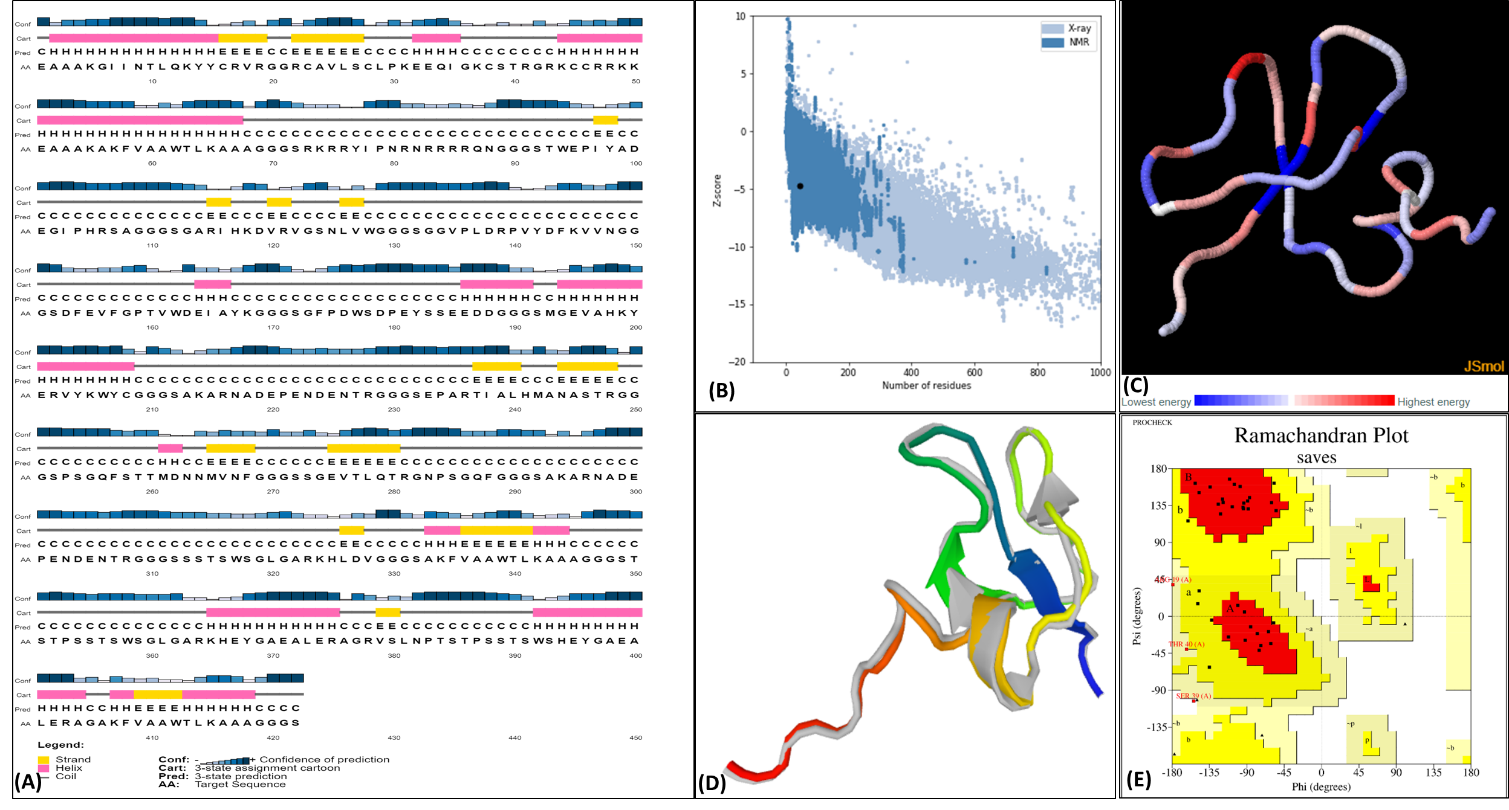


**Figure S6:** 2D and 3D structure refinement and validation of MLB1-C2 (A) secondary structure of MLB1-C2 construct (B) the protein's overall quality, indicated by the Z-score of -4.71, (C) Tertiary structure energy map (D) Designed MLB1-C2 refined structure (E) The refined vaccine protein displayed outstanding structural characteristics with 73.7% of residues in highly favored regions, 18.4% in the allowed region, generously allowed (5.3%), and disallowed (2.6%) regions in the Ramachandran plot.


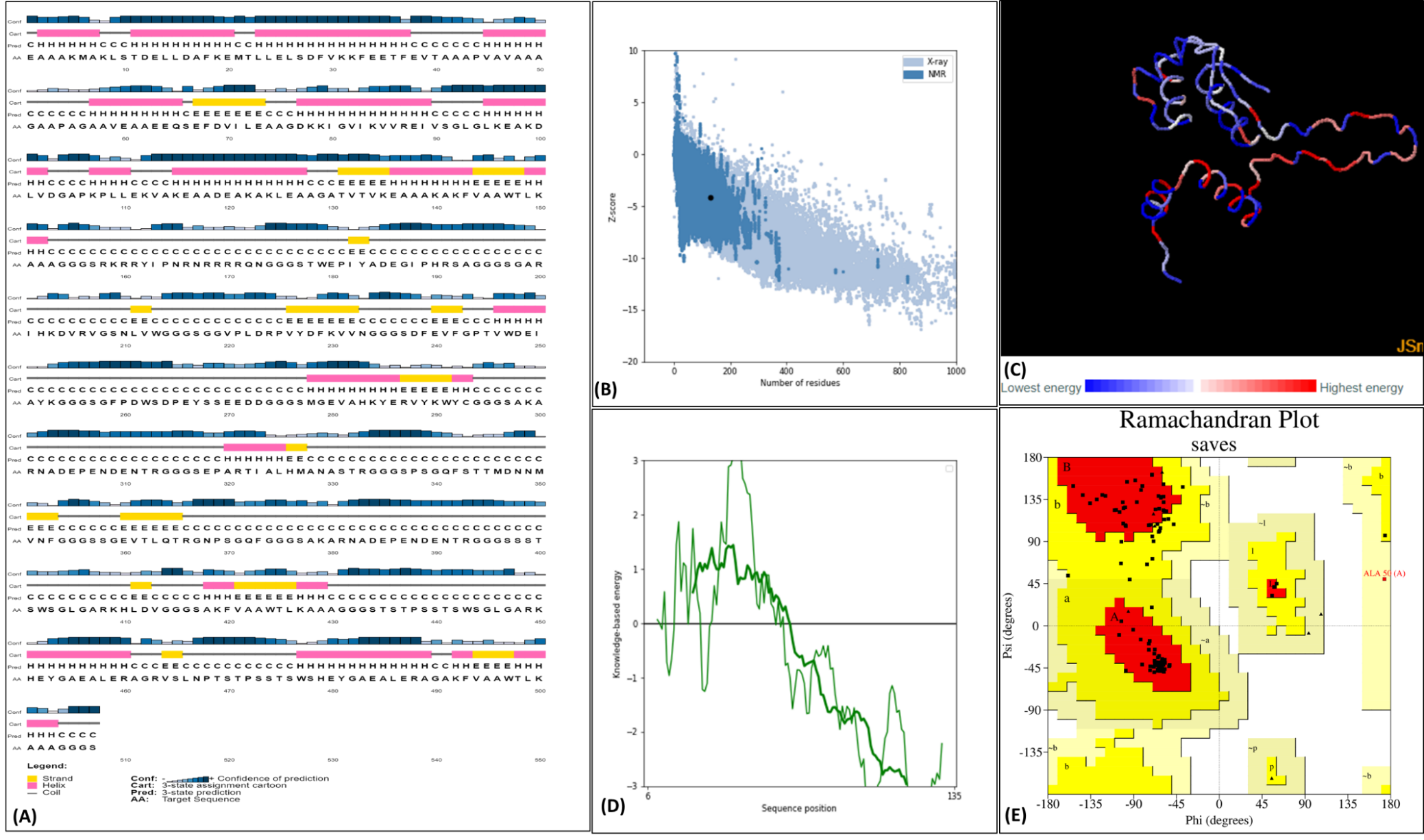


**Figure S7:** 2D and 3D structure refinement and validation of MLB1-C2 (A) secondary structure of MLB1-C2 construct (B) the protein's overall quality, indicated by the Z-score of -4.11, (C) Tertiary structure energy map (D) ProSA-web plot showing residue scores in a native protein structure (E) The refined vaccine protein displayed outstanding structural characteristics with 82.8% of residues in highly favored regions, 16.4% in the allowed region, generously allowed (0.9%), and disallowed (0.0%) regions in the Ramachandran plot.


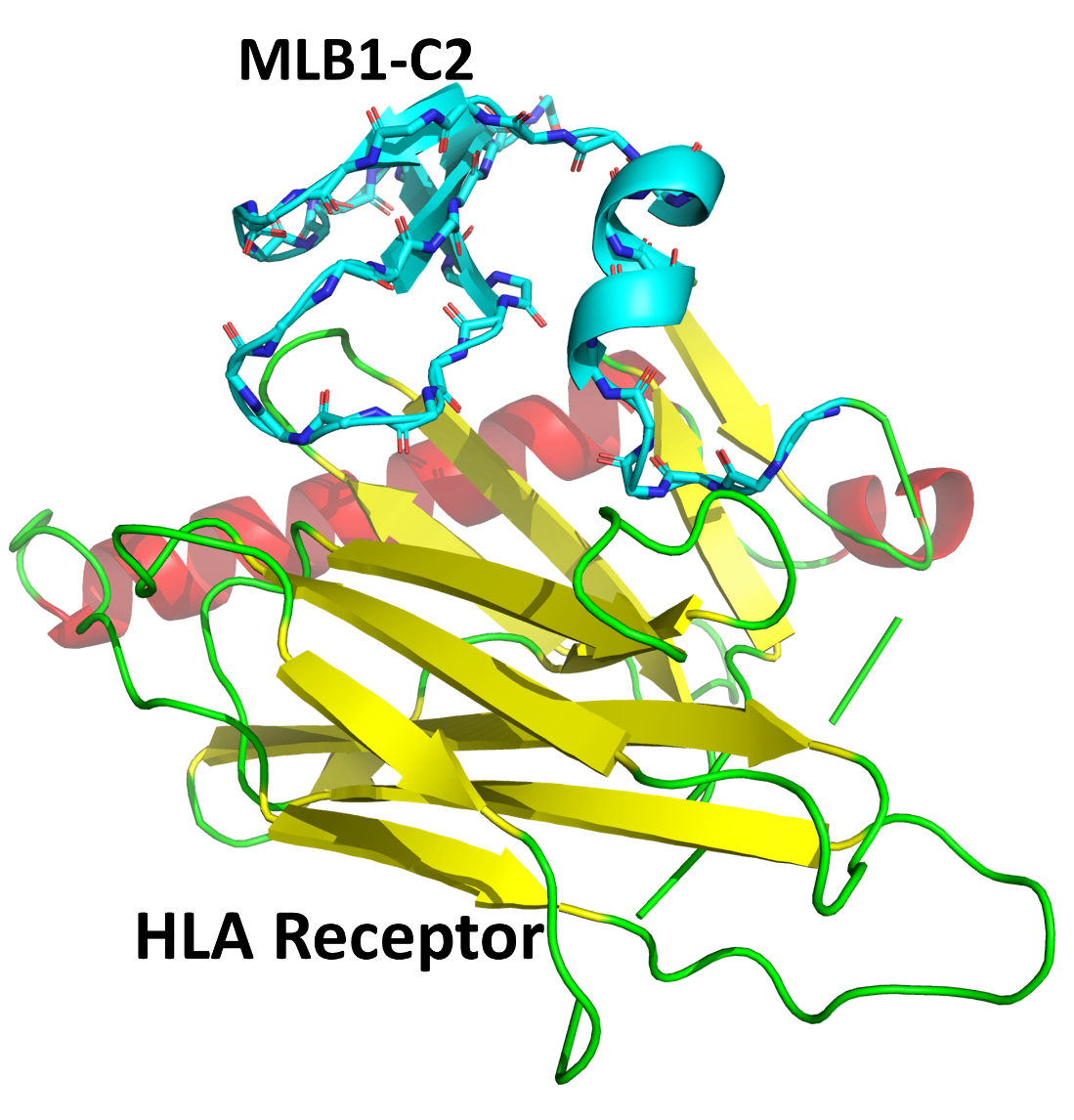


**Figure S7:** The structural representation of the most populated cluster of the vaccine V2 and HLA receptor complex during a 100 ns molecular dynamics simulation is shown. The complex is depicted in cartoon form, with vaccine V2 colored in cyan, and the receptor HLA highlighted in green, red, and yellow


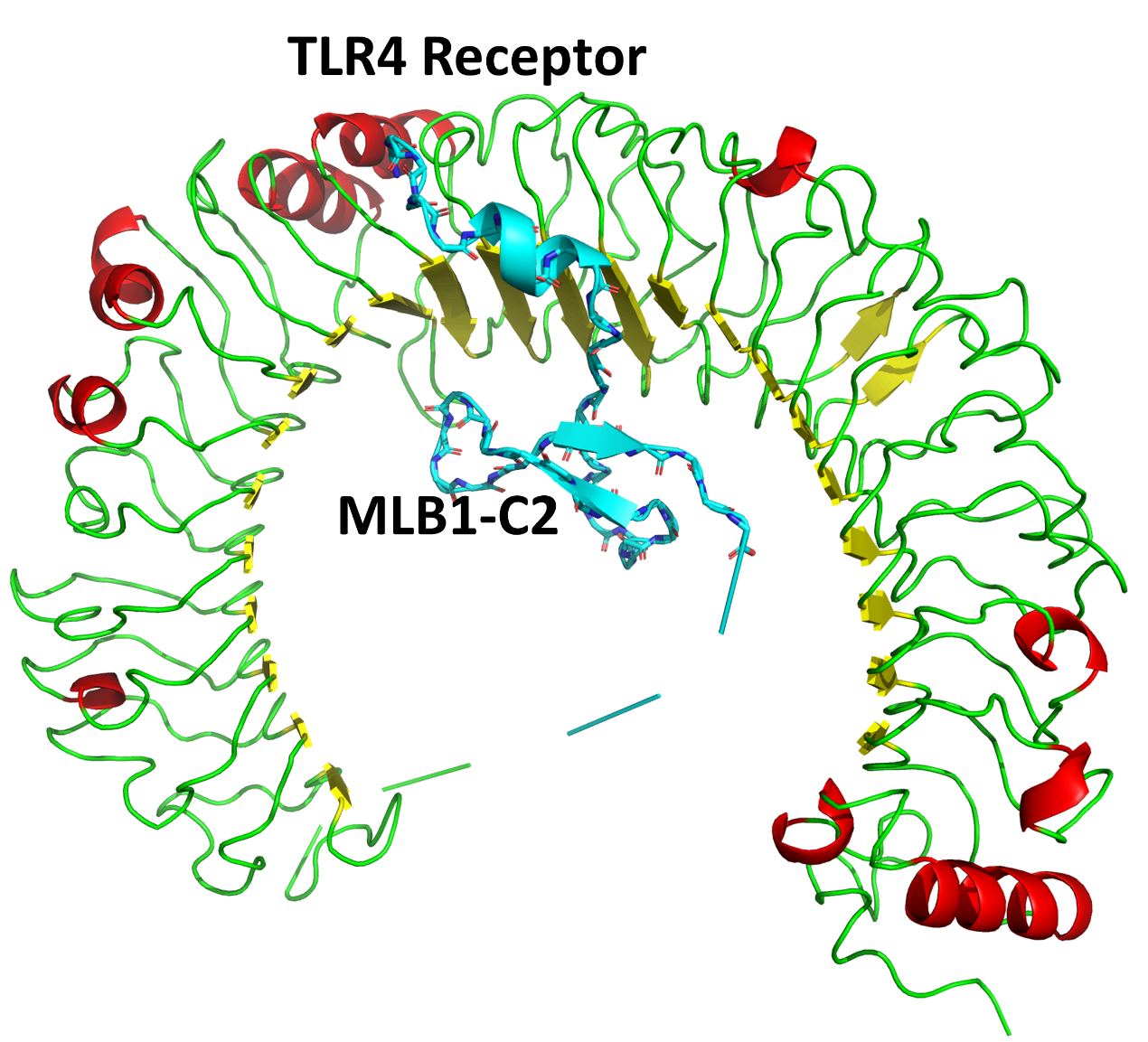


**Figure S8:** The structural representation of the most populated cluster of the vaccine V2 and TLR4 receptor complex during a 100 ns molecular dynamics simulation is shown. The complex is depicted in cartoon form, with vaccine V2 colored in cyan, and the receptor TLR4 highlighted in green, red, and yellow.


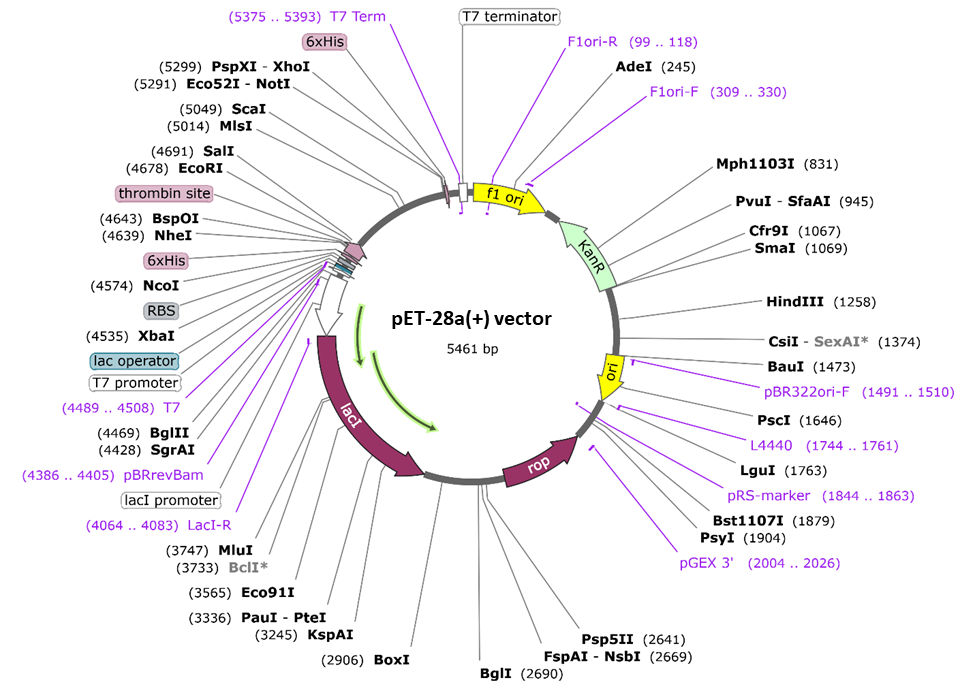


**Figure S9:** *In-silico* cloning of lead vaccine construct (MLB1-V2) in pET-28a(+) vector.

**Supplementary Figure S10:** Signal peptide calculation in the vaccine construct MLB1-C1.


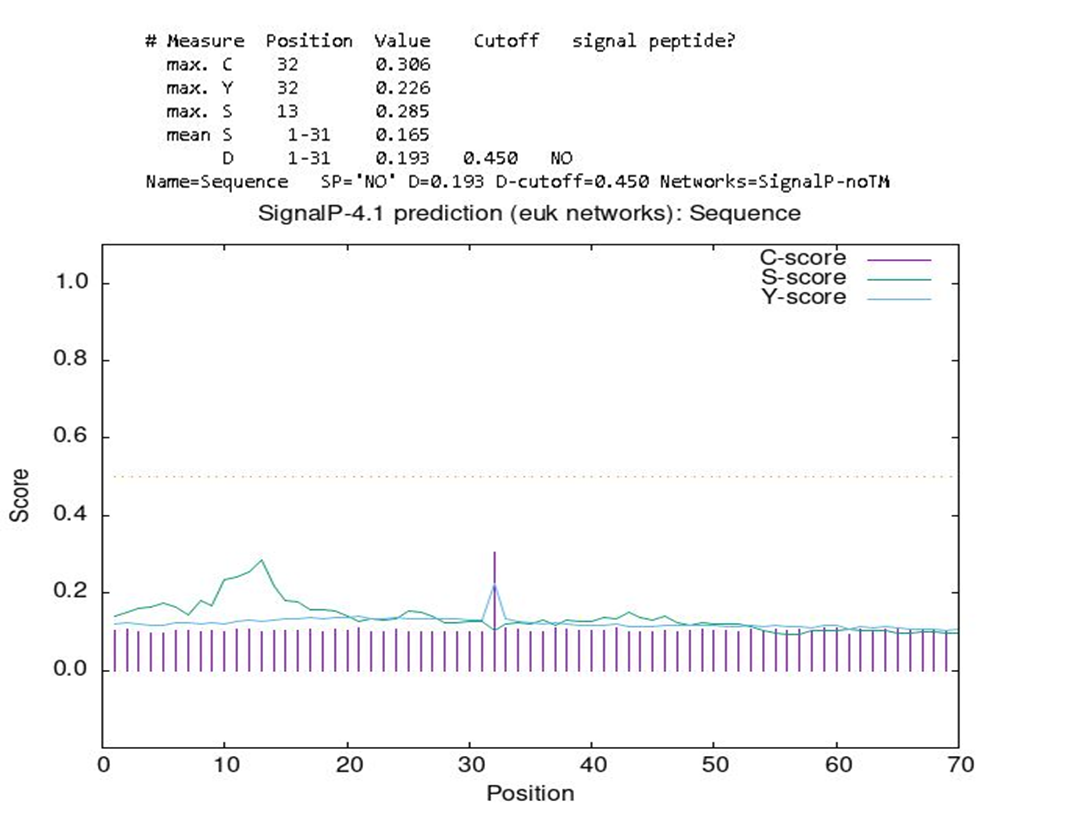


**Supplementary Figure S11:** Signal peptide calculation in the vaccine construct MLB1-C2.

**
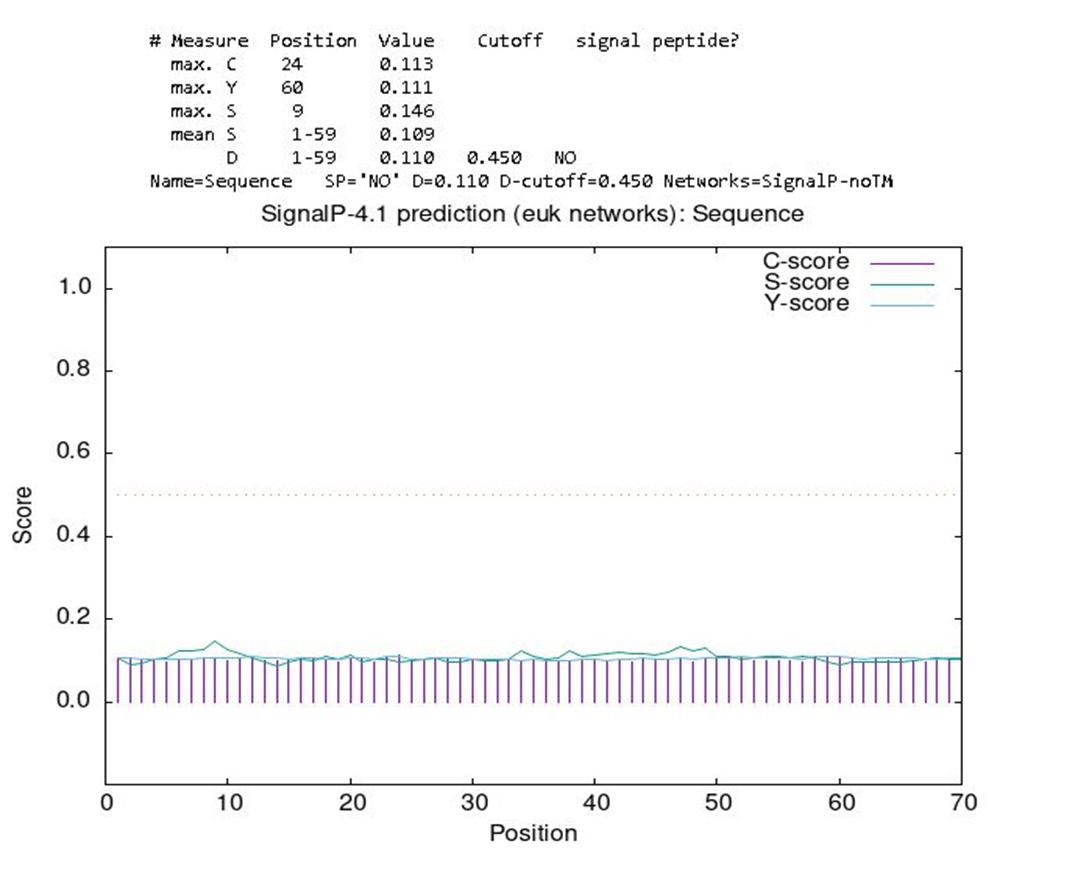
**

**Supplementary Figure S12:** Signal peptide calculation in the vaccine construct MLB1-C3.

**
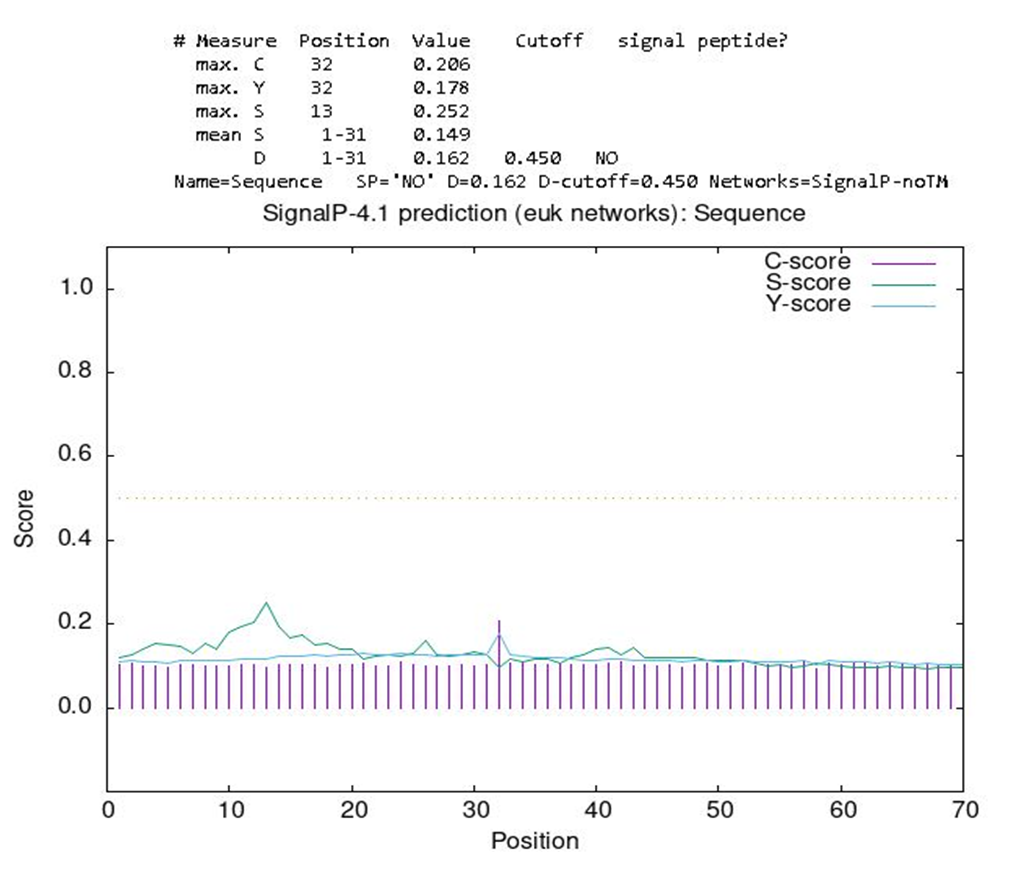
**

**Supplementary Figure S13:** Signal peptide calculation in the vaccine construct MLB1-C4.

**
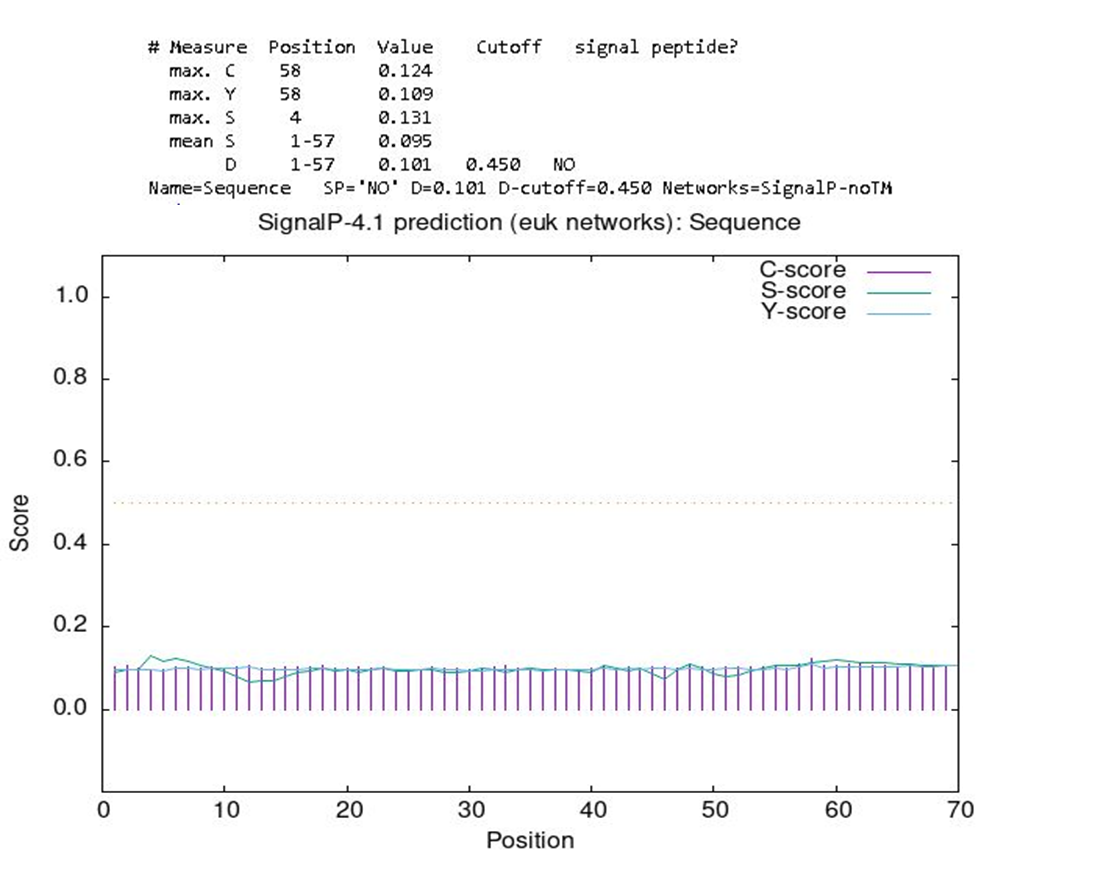
**

**Supplementary Figure S14:** Transmembrane helix calculation in the vaccine construct MLB1-C1.


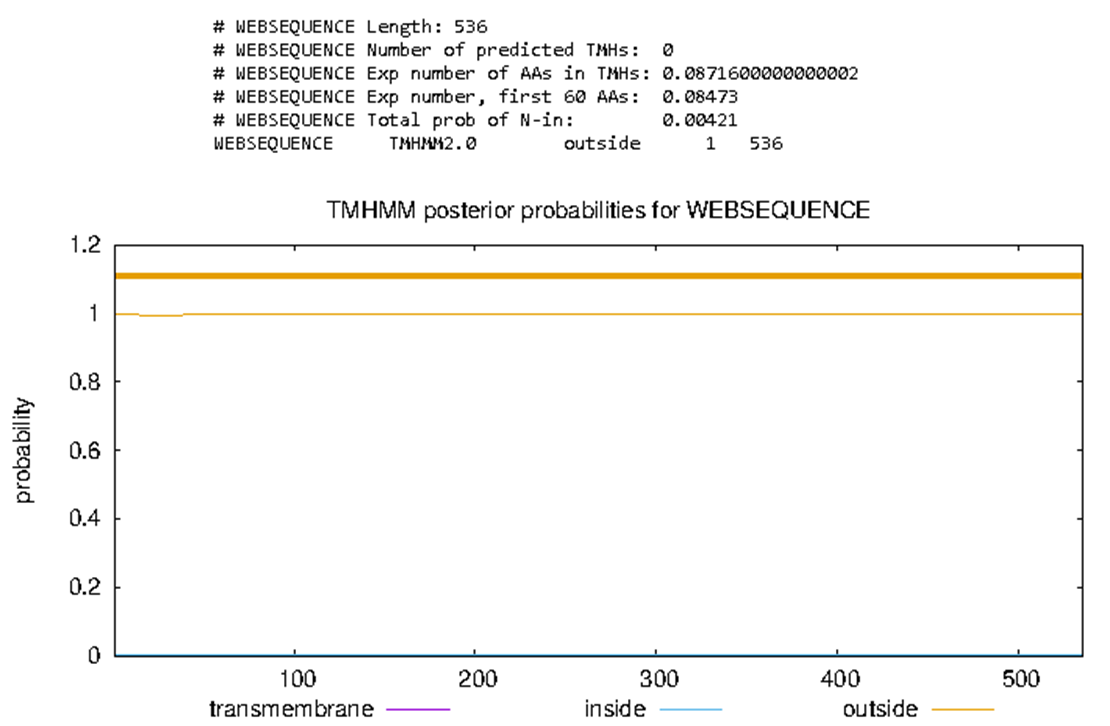


**Supplementary Figure S15:** Transmembrane helix calculation in the vaccine construct MLB1-C2.


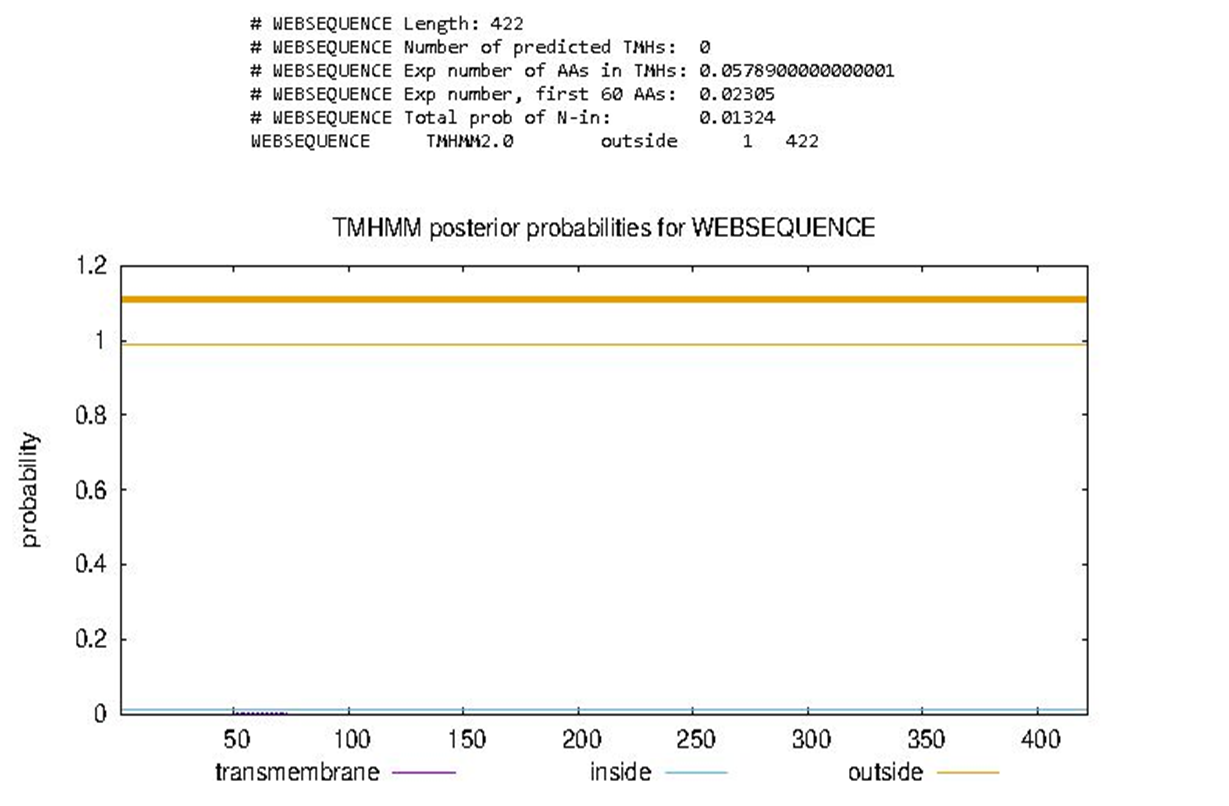


**Supplementary Figure S16:** Transmembrane helix calculation in the vaccine construct MLB1-C3.


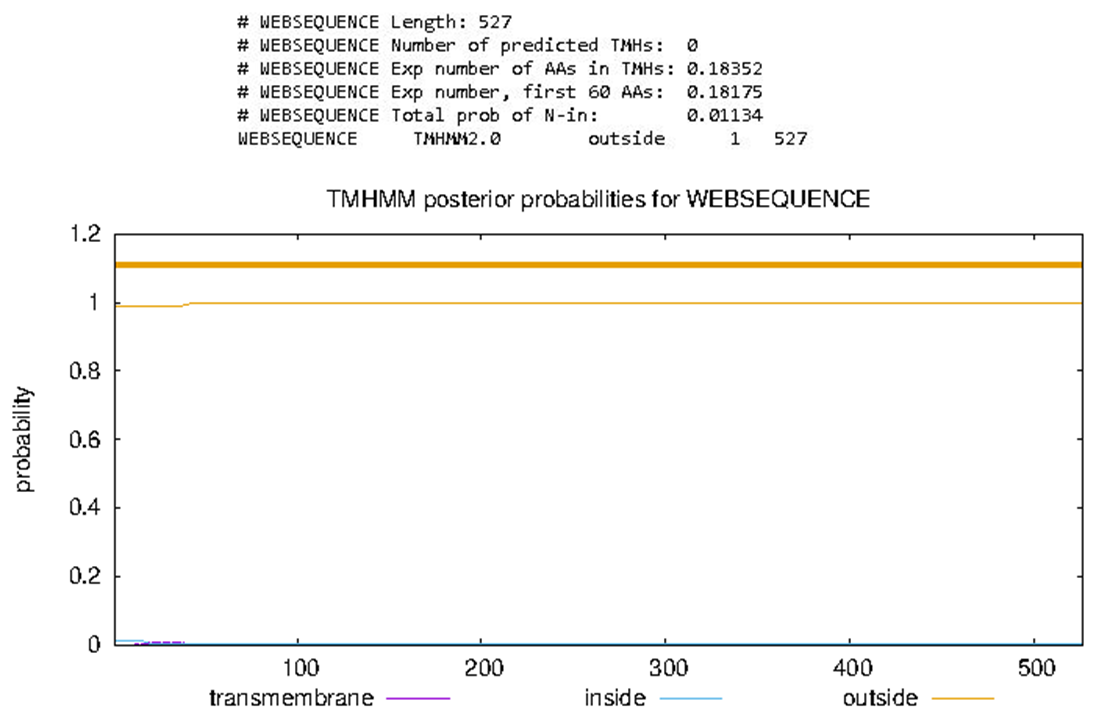


**Supplementary Figure S17:** Transmembrane helix calculation in the vaccine construct MLB1-C4.


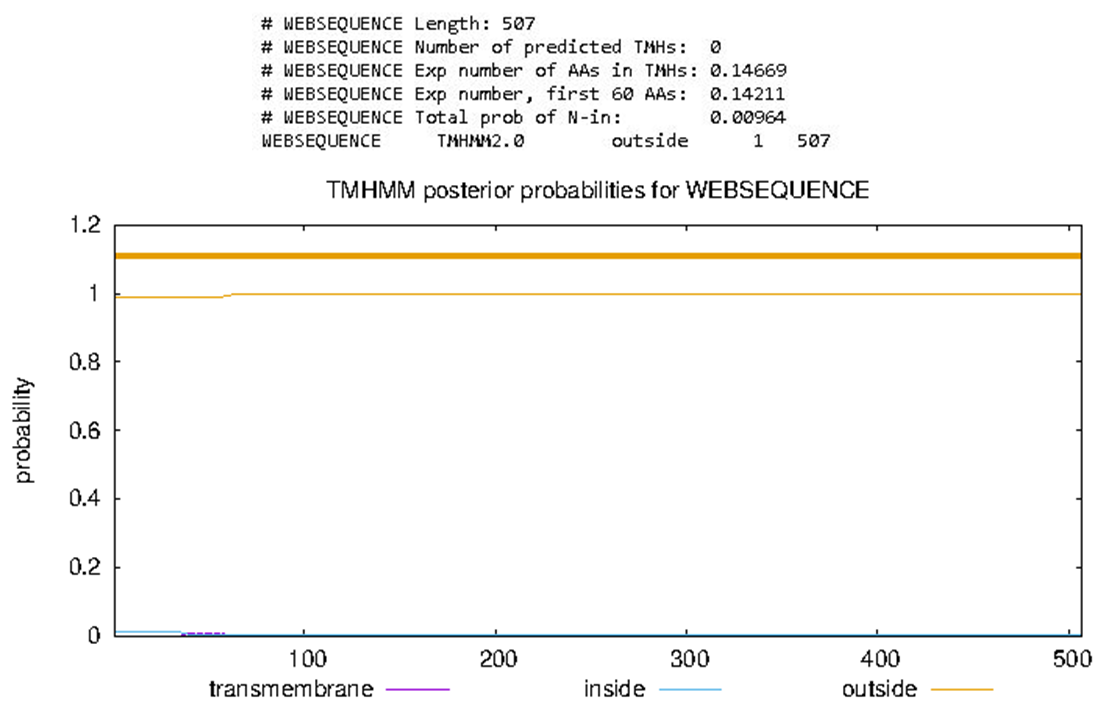


**Supplementary Tables:**

**Table S1**. The top-ranked proteins for vaccine candidate of MLB1 for designing MEV constructs.

| **Proteins Ids** | **Allergenicity** | **Toxicity** | **Antigenicity** |
| --- | --- | --- | --- |
| BAU68081.1 | Non-allergen | Non-toxic | 0.4484 |
| QQM16406.1 | Non-allergen | Non-toxic | 0.4543 |
| AKA09822.1 | Non-allergen | Non-toxic | 0.5197 |
| AKA09787.1 | Non-allergen | Non-toxic | 0.4480 |
| AFJ68608.1 | Non-allergen | Non-toxic | 0.7579 |

**Table S2:** Prioritized B-Cell and T-cell epitopes overlapping.

| **Protein IDs** | **MHC-I Epitopes** | **IC_50_** | **MHC-II Epitopes** | **IC_50_** | **B-Cell Epitopes** |
| --- | --- | --- | --- | --- | --- |
| BAU68081.1 | APKFRKRRY | 6.8 | KRRYIPNRNRRRRQN | 33.3 | RKRRYIPNRNRRRRQN |
|  | TWEPIYADE | 18.0 | EPIYADEGIPHRSAL | 95.6 | TWEPIYADEGIPHRSA |
|  | GARIHKDVR | 13.3 | IHKDVRVGSNLVWRI | 19.2 | GARIHKDVRVGSNLVW |
| QQM16406.1 MAG | LYGGVPLDR | 14.5 | VYDFKVVNEDLLGLL | 33.8 | GGVPLDRPVYDFKVVN |
|  | NNDFEVFGP | 7.3 | VWDEIAYKKSFEKFE | 38.0 | DFEVFGPTVWDEIAYK |
|  | IGFPDWSDP | 16.6 | DAIEEIVRARIGFPD | 43.0 | GFPDWSDPEYSSEEDD |
| AKA09822.1 | MGEVAHKYE | 37.5 | ERVYKWYCRNLVNRF | 89.2 | MGEVAHKYERVYKWYC |
|  | VYKWYCRNL | 8.2 | RNLVNRFVILPSGEV | 41.6 | AKARNADEPENDENTR |
|  | HFDTIVYGD | 8.8 | EDEWIHFDTIVYGDD | 24.3 | EPARTIALHMANASTR |
| AKA09787.1 | NPSGQFSTT | 9.5 | SGQFSTTMDNNMVNF | 38.9 | PSGQFSTTMDNNMVNF |
|  | SGEVTLQTR | 24.8 | GNPSGQFSTTMDNNM | 42.2 | SGEVTLQTRGNPSGQF |
|  | INKDQREKY | 4.6 | RHVLLPSGEVTLQTR | 14.9 | AKARNADEPENDENTR |
| AFJ68608.1 | SSTSWSGLG | 46.0 | WNFINKDQREKYRHV | 27.3 | SSTSWSGLGARKHLDV |
|  | TSTPSSTSW | 5.4 | NFINKDQREKYRHVH | 27.7 | TSTPSSTSWSGLGARK |
|  | RVSLNPTST | 35.4 | DTVVYGDDRLSTTPS | 33.8 | RVSLNPTSTPSSTSWS |

**Table S3:** considered human HLA ‏ for the study of MHC class II and MHC class I.

| HLA alleles |  |
| --- | --- |
| MHC-I | HLA-A*01:01,HLA-A*02:01,HLA-A*02:06,HLA-A*03:01,HLA-A*11:01,HLA-A*23:01,HLA-A*24:02,HLA-A*25:01,HLA-A*26:01,HLA-A*29:02,HLA-A*30:01,HLA-A*30:02,HLA-A*31:01,HLA-A*32:01,HLA-A*68:01,HLA-A*68:02,HLA-B*07:02,HLA-B*08:01,HLA-B*14:02,HLA-B*15:01,HLA-B*15:02,HLA-B*18:01,HLA-B*27:05,HLA-B*35:01,HLA-B*35:03,HLA-B*35:03,HLA-B*38:01,HLA-B*39:01,HLA-B*40:01,HLA-B*40:02,HLA-B*44:03,HLA-B*46:01,HLA-B*48:01,HLA-B*51:01,HLA-B*53:01,HLA-B*57:01,HLA-B*58:01,HLA-B*58:02,HLA-C*03:03,HLA-C*04:01,HLA-C*05:01,HLA-C*06:02,HLA-C*07:01,HLA-C*07:02,HLA-C*08:02,HLA-C*12:03,HLA-C*14:02,HLA-C*14:02,HLA-C*15:02,HLA-E*01:01,HLA-E*01:03 |
| MHC-II | DRB1*01,DRB1*03,DRB1*04,DRB1*04,DRB1*04,DRB1*07,DRB1*08,DRB1*09,DRB1*11,DRB1*12,DRB1*13, DRB1*15,DRB3*01,DRB4*01,DRB5*01,DRB1*01,DRB1*03,DRB1*04,DRB1*04,DRB1*04, DRB1*07,DRB1*08,DRB1*09, DRB1*11,DRB1*12,DRB1*13,DRB1*15,DRB3*01,DRB4*01,DRB5*01,DRB1*01,DRB1*03,DRB1*04,DRB1*04,  DRB1*04,DRB1*07,DRB1*08,DRB1*09,DRB1*11,DRB1*12, DRB1*13,DRB1*15,DRB3*01,DRB4*01,DRB5*01. |

**Table S4:** Population coverage of T-cell epitopes across different human ethnicities.

| Proteins | BAU68081.1* | | QQM16406.1 MAG* | | AKA09822.1* | | AKA09787.1* | | AKA09787.1* | |
| --- | --- | --- | --- | --- | --- | --- | --- | --- | --- | --- |
| Coverage | MHC-1 | MHC-2 | MHC-1 | MHC-2 | MHC-1 | MHC-2 | MHC-1 | MHC-2 | MHC-1 | MHC-2 |
| Central Africa | 98.39% | 49.02% | 96.02% | 58.02% | 86.50% | 98.39% | 92.64% | 38.55% | 98.42% | 62.84% |
| Central America | 9.07% | 29.86% | 9.07% | 32.91% | 3.56% | 9.07% | 7.01% | 18.53% | 10.30% | 53.91% |
| East Africa | 98.74% | 53.35% | 96.33% | 62.42% | 88.28% | 98.74% | 93.07% | 48.16% | 98.79% | 68.53% |
| East Asia | 100.0% | 73.63% | 99.51% | 76.00% | 95.83% | 100.00% | 91.31% | 27.52% | 100.00% | 82.41% |
| Europe | 100.0% | 75.87% | 99.88% | 85.71% | 98.16% | 100.00% | 97.36% | 61.97% | 100.00% | 87.47% |
| North Africa | 99.60% | 61.73% | 98.50% | 74.88% | 94.79% | 99.60% | 95.88% | 53.97% | 99.61% | 76.07% |
| Northeast Asia | 98.55% | 78.65% | 99.65% | 87.96% | 96.64% | 98.55% | 95.80% | 56.87% | 100.00% | 60.80% |
| Oceania | 99.15% | 51.23% | 97.84% | 55.60% | 93.47% | 99.15% | 83.54% | 22.97% | 98.59% | 60.21% |
| South Africa | 99.64% | 51.30% | 97.96% | 52.15% | 94.56% | 99.64% | 81.41% | 23.06% | 99.64% | 32.10% |
| South America | 100.0% | 7.65% | 98.99% | 32.10% | 96.56% | 100.00% | 96.81% | 25.52% | 100.00% | 63.52% |
| South Asia | 100.0% | 41.65% | 96.22% | 48.29% | 88.02% | 100.00% | 86.18% | 25.81% | 100.00% | 76.44% |
| Southeast Asia | 100.0% | 65.03% | 97.27% | 74.42% | 90.86% | 100.00% | 85.31% | 24.27% | 100.00% | 58.83% |
| Southwest Asia | 98.66% | 50.14% | 98.47% | 55.72% | 93.17% | 98.66% | 83.10% | 30.92% | 98.70% | 45.29% |
| West Africa | 99.12% | 36.92% | 96.10% | 44.26% | 88.83% | 99.12% | 87.56% | 33.53% | 99.16% | 65.81% |
| West Indies | 99.57% | 57.03% | 97.34% | 63.10% | 89.24% | 99.57% | 93.54% | 46.81% | 99.61% | 70.02% |
| World | 100.0% | 60.98% | 98.53% | 65.55% | 96.63% | 100.00% | 94.90% | 51.41% | 100.00% | 83.81% |

*BAU68081.1 putative capsid protein.

*QQM16406.1 MAG: nonstructural protein.

*AKA09822.1 RNA-dependent RNA polymerase, partial.

*AKA09787.1 RNA-dependent RNA polymerase, partial.

*AFJ68608.1 capsid, partial.

**Table S5:** The designed four vaccines anti-HAstV-MLB1 constructs with appropriate adjuvants and linkers.

| **Vaccine constructs with Adjuvant** | **Construct** | **Allergenicity** | **Antigenpro** | **Toxocity** | **Solubility** |
| --- | --- | --- | --- | --- | --- |
| **MLB1-C1 with HBHA adjuvant** | EAAAKMAENPNIDDLPAPLLAALGAADLALATVNDLIANLRERAEETRAETRTRVEERRARLTKFQEDLPEQFIELRDKFTTEELRKAAEGYLEAATNRYNELVERGEAALQRLRSQTAFEDASARAEGYVDQAVELTQEALGTVASQTRAVGERAAKLVGIELEAAAKAKFVAAWTLKAAAGGGSRKRRYIPNRNRRRRQNGGGSTWEPIYADEGIPHRSAGGGSGARIHKDVRVGSNLVWGGGSGGVPLDRPVYDFKVVNGGGSDFEVFGPTVWDEIAYKGGGSGFPDWSDPEYSSEEDDGGGSMGEVAHKYERVYKWYCGGGSAKARNADEPENDENTRGGGSEPARTIALHMANASTRGGGSPSGQFSTTMDNNMVNFGGGSSGEVTLQTRGNPSGQFGGGSAKARNADEPENDENTRGGGSSSTSWSGLGARKHLDVGGGSAKFVAAWTLKAAAGGGSTSTPSSTSWSGLGARKHEYGAEALERAGRVSLNPTSTPSSTSWSHEYGAEALERAGAKFVAAWTLKAAAGGGS | PROBABLE NON-ALLERGEN | 0.6312 ( Probable ANTIGEN ). | Non-Toxin | 0.586 |
| **MLB1-C2 with β-definsin adjuvant** | EAAAKMAENPNIDDLPAPLLAALGAADLALATVNDLIANLRERAEETRAETRTRVEERRARLTKFQEDLPEQFIELRDKFTTEELRKAAEGYLEAATNRYNELVERGEAALQRLRSQTAFEDASARAEGYVDQAVELTQEALGTVASQTRAVGERAAKLVGIELEAAAKAKFVAAWTLKAAAGGGSRKRRYIPNRNRRRRQNGGGSTWEPIYADEGIPHRSAGGGSGARIHKDVRVGSNLVWGGGSGGVPLDRPVYDFKVVNGGGSDFEVFGPTVWDEIAYKGGGSGFPDWSDPEYSSEEDDGGGSMGEVAHKYERVYKWYCGGGSAKARNADEPENDENTRGGGSEPARTIALHMANASTRGGGSPSGQFSTTMDNNMVNFGGGSSGEVTLQTRGNPSGQFGGGSAKARNADEPENDENTRGGGSSSTSWSGLGARKHLDVGGGSAKFVAAWTLKAAAGGGSTSTPSSTSWSGLGARKHEYGAEALERAGRVSLNPTSTPSSTSWSHEYGAEALERAGAKFVAAWTLKAAAGGGS | PROBABLE NON-ALLERGEN | 0.6707 ( Probable ANTIGEN ). | Non-Toxin | 0.874 |
| **MLB1-C3 with HBHA conserved adjuvant** | EAAAKMAENSNIDDIKAPLLAALGAADLALATVNELITNLRERAEETRRSRVEESRARLTKLQEDLPEQLTELREKFTAEELRKAAEGYLEAATSELVERGEAALERLRSQQSFEEVSARAEGYVDQAVELTQEALGTVASQVEGRAAKLVGIELEAAAKAKFVAAWTLKAAAGGGSRKRRYIPNRNRRRRQNGGGSTWEPIYADEGIPHRSAGGGSGARIHKDVRVGSNLVWGGGSGGVPLDRPVYDFKVVNGGGSDFEVFGPTVWDEIAYKGGGSGFPDWSDPEYSSEEDDGGGSMGEVAHKYERVYKWYCGGGSAKARNADEPENDENTRGGGSEPARTIALHMANASTRGGGSPSGQFSTTMDNNMVNFGGGSSGEVTLQTRGNPSGQFGGGSAKARNADEPENDENTRGGGSSSTSWSGLGARKHLDVGGGSAKFVAAWTLKAAAGGGSTSTPSSTSWSGLGARKHEYGAEALERAGRVSLNPTSTPSSTSWSHEYGAEALERAGAKFVAAWTLKAAAGGGS | PROBABLE NON-ALLERGEN | 0.6299 ( Probable ANTIGEN ). | Non-Toxin | 0.622 |
| **MLB1-C4 with Ribosomal protein adjuvant** | EAAAKMAKLSTDELLDAFKEMTLLELSDFVKKFEETFEVTAAAPVAVAAAGAAPAGAAVEAAEEQSEFDVILEAAGDKKIGVIKVVREIVSGLGLKEAKDLVDGAPKPLLEKVAKEAADEAKAKLEAAGATVTVKEAAAKAKFVAAWTLKAAAGGGSRKRRYIPNRNRRRRQNGGGSTWEPIYADEGIPHRSAGGGSGARIHKDVRVGSNLVWGGGSGGVPLDRPVYDFKVVNGGGSDFEVFGPTVWDEIAYKGGGSGFPDWSDPEYSSEEDDGGGSMGEVAHKYERVYKWYCGGGSAKARNADEPENDENTRGGGSEPARTIALHMANASTRGGGSPSGQFSTTMDNNMVNFGGGSSGEVTLQTRGNPSGQFGGGSAKARNADEPENDENTRGGGSSSTSWSGLGARKHLDVGGGSAKFVAAWTLKAAAGGGSTSTPSSTSWSGLGARKHEYGAEALERAGRVSLNPTSTPSSTSWSHEYGAEALERAGAKFVAAWTLKAAAGGGS | PROBABLE NON-ALLERGEN | 0.6123 ( Probable ANTIGEN ). | Non-Toxin | 0.520 |

**Table S6:** Secondary structure composition of β-strands, and α-helices motifs in vaccine constructs

| S.No | Vaccine constructs | β-strands | α-helices |
| --- | --- | --- | --- |
| 01 | MLB1-C1 | 26 Amino acid | 222 Amino acid |
| 02 | MLB1-C2 | 29 Amino acid | 198 Amino acid |
| 03 | MLB1-C3 | 31 Amino acid | 232 Amino acid |
| 04 | MLB1-C4 | 59 Amino acid | 170 Amino acid |
